# Blautia Coccoides is a Newly Identified Bacterium Increased by Leucine Deprivation and has a Novel Function in Improving Metabolic Disorders

**DOI:** 10.1002/advs.202309255

**Published:** 2024-03-01

**Authors:** Yuguo Niu, Xiaoming Hu, Yali Song, Cunchuan Wang, Peixiang Luo, Shihong Ni, Fuxin Jiao, Ju Qiu, Weihong Jiang, Sheng Yang, Jun Chen, Rui Huang, Haizhou Jiang, Shanghai Chen, Qiwei Zhai, Jia Xiao, Feifan Guo

**Affiliations:** ^1^ Zhongshan Hospital State Key Laboratory of Medical Neurobiology Institute for Translational Brain Research MOE Frontiers Center for Brain Science Fudan University Shanghai 200032 China; ^2^ Department of Metabolic and Bariatric Surgery and Clinical Research Institute First Affiliated Hospital of Jinan University Guangzhou 510632 China; ^3^ CAS Key Laboratory of Nutrition Metabolism and Food Safety Innovation Center for Intervention of Chronic Disease and Promotion of Health Shanghai Institute of Nutrition and Health University of Chinese Academy of Sciences Chinese Academy of Sciences Shanghai 200031 China; ^4^ Key Laboratory of Synthetic Biology Institute of Plant Physiology and Ecology CAS Center for Excellence in Molecular Plant Science Shanghai 200032 China

**Keywords:** aryl hydrocarbon receptor, blautia coccoides, gut microbiota, indole‐3‐acetic acid, leucine deprivation

## Abstract

Gut microbiota is linked to human metabolic diseases. The previous work showed that leucine deprivation improved metabolic dysfunction, but whether leucine deprivation alters certain specific species of bacterium that brings these benefits remains unclear. Here, this work finds that leucine deprivation alters gut microbiota composition, which is sufficient and necessary for the metabolic improvements induced by leucine deprivation. Among all the affected bacteria, *B. coccoides* is markedly increased in the feces of leucine‐deprived mice. Moreover, gavage with *B. coccoides* improves insulin sensitivity and reduces body fat in high‐fat diet (HFD) mice, and singly colonization of *B. coccoides* increases insulin sensitivity in gnotobiotic mice. The effects of *B. coccoides* are mediated by metabolizing tryptophan into indole‐3‐acetic acid (I3AA) that activates the aryl hydrocarbon receptor (AhR) in the liver. Finally, this work reveals that reduced fecal *B. coccoides* and I3AA levels are associated with the clinical metabolic syndrome. These findings suggest that *B. coccoides* is a newly identified bacterium increased by leucine deprivation, which improves metabolic disorders via metabolizing tryptophan into I3AA.

## Graphical abstract

Leucine deprivation increases the abundance of *B. coccoides*, which improves insulin sensitivity and reduces body fat in HFD mice by metabolizing tryptophan into I3AA that activates AhR in the liver.

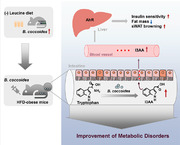

## 1 Introduction

Obesity and related metabolic disorders, including type 2 diabetes, have become the most prevalent public health concerns worldwide, leading to global epidemics.^[^ ^1^ ^]^ Over the past two decades, accumulated data from genetic analyses have demonstrated the associations between gut microbiota composition and metabolic diseases.^[^ ^2^, ^3^, ^4^ ^]^ Moreover, studies indicated that germ‐free mice had reduced adiposity, which was reversed by colonization with a normal gut microbiota.^[^ ^5^, ^6^ ^]^ Studies further indicated that germ‐free mice developed obese phenotype after receiving fecal microbiota from obese individuals.^[^ ^7^, ^8^ ^]^ Gut microbiota is considered as the second human genome and its interaction with the host is complex. Bacterial cellular components^[^ ^9^ ^]^ and metabolites^[^ ^10^, ^11^, ^12^ ^]^ contributed to the regulation of host physiology and the maintenance of metabolic homeostasis. These studies suggested that regulation of intestinal microbiome could prevent or treat metabolic diseases.

Among the factors that modulate gut microbiota, host diet has a profound impact.^[^ ^13^, ^14^ ^]^ For example, dysbiosis of gut microbiota induced by high‐fat/high‐calorie diet promoted the development of obesity, insulin resistance, and other hallmarks of metabolic syndromes.^[^ ^3^, ^15^, ^16^ ^]^ In contrast, administration of a short‐term diet containing indigestible carbohydrates to a subgroup of healthy humans reshaped their gut microbiota, including the increase of *Prevotella*, which provided beneficial effects to the metabolic regulation.^[^ ^17^ ^]^ Recent studies indicated that the variation of protein content in the diet could change the gut microbial composition. For example, protein consumption was positively correlated with high microbial diversity.^[^ ^18^ ^]^ Interestingly, a recent study reported that the gut symbiont *Clostridium sporogenes* metabolized aromatic amino acids (including tryptophan, phenylalanine, and tyrosine) to produce twelve compounds, one of which is indole‐3‐propionic acid,^[^ ^19^ ^]^ which played a key role in axonal regeneration.^[^ ^20^ ^]^ To date, few studies have investigated the roles and the underlying mechanisms of specific species of intestinal bacteria in regulating metabolic homeostasis.^[^ ^12^, ^17^, ^21^, ^22^ ^]^ The mechanisms by which gut microbiota respond to amino acid changes and the causal relationship between specific bacteria and metabolic diseases remain unclear.

Amino acids can regulate metabolic processes. Accumulated studies indicated that increased circulating branched‐chain amino acid (BCAA, including leucine, isoleucine, and valine) levels were associated with a higher risk of obesity, insulin resistance, type 2 diabetes, and other complications of metabolic syndrome in mice and humans.^[^ ^23^ ^]^ Consequently, many recent studies indicated that reducing the dietary supply of BCAAs improves glucose tolerance and other metabolic endpoints.^[^ ^24^ ^]^ Previous studies indicated that leucine deprivation dramatically improved insulin sensitivity^[^ ^25^, ^26^ ^]^ and reduced body weight and fat mass.^[^ ^27^ ^]^ As gut microbiota has been reported to associate with metabolic homeostasis and can be altered by nutrition, we hypothesize that gut microbiota modulated by leucine deprivation may provide some metabolic benefits. Hence, we try to explore which specific intestinal bacterium altered by leucine deprivation can bring benefits to the host metabolism.

## 2 Results

### 2.1 The Gut Microbiota Modified by Leucine Deprivation are Sufficient and Necessary for the Metabolic Improvements

**Figure 1:**
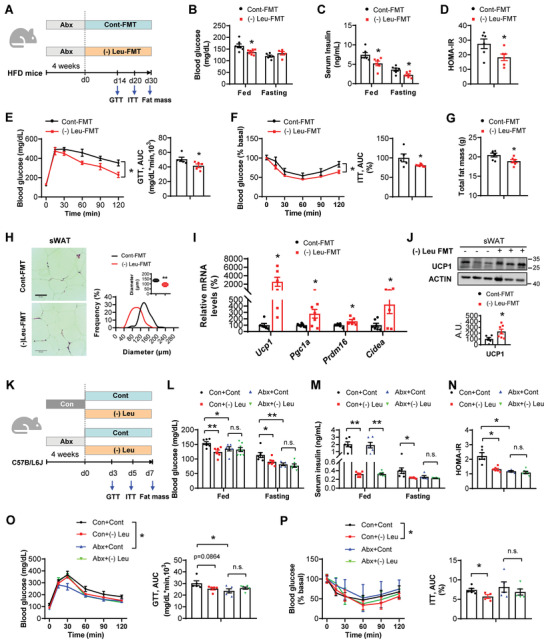
The gut microbiota modified by leucine deprivation is sufficient and necessary for the metabolic improvements. A–J) HFD mice were given with antibiotic cocktail (termed as Abx) in drinking water. After 4 weeks, the Abx mice were transplanted with the fecal microbiota from control diet (Cont‐FMT) or leucine‐deprived diet ((‐) Leu‐FMT) donors daily for 30 days (*n* = 5–7 biological replicates per group). A) Experimental procedures. B) Fed and fasting blood glucose levels. C) Fed and fasting serum insulin levels assayed by ELISA. D) HOMA‐IR index. E) Glucose tolerance tests. The right panel is the area under curve (AUC). F) Insulin tolerance tests (1 U kg^−1^). The right panel is the AUC. G) Total fat mass. H) The H&E staining of subcutaneous white adipose tissue (sWAT). Scale bars, 50 µm. The right panel is the frequency distribution of adipocyte cell size in sWAT and the box plot is the average adipocyte diameter. I) Real‐time PCR analysis of browning related genes (*Ucp1*, *Pgc1α*, *Prdm16*, and *Cidea*) in sWAT. J) Western blot analysis of UCP1 protein levels in sWAT. The bottom panel is the densitometry analysis of UCP1 protein levels. A.U.: arbitrary units. K–P) 10‐week‐old male C57BL/6J wild‐type (WT) mice were given Abx in drinking water or given autoclaved water (Con). After 4 weeks, the Con or Abx mice were treated with the (‐) Leu or Cont diet for 7 days, respectively (*n* = 5–8 biological replicates per group). K) Experimental procedures. L) Fed and fasting blood glucose levels. M) Fed and fasting serum insulin levels assayed by ELISA. N) HOMA‐IR index. O) Glucose tolerance tests. The right panel is the AUC. P) Insulin tolerance tests (0.5 U kg^−1^). The right panel is the AUC. All values are expressed as the mean ± SEM. Statistical comparisons were carried out by unpaired two‐tailed Student's *t*‐test or two‐way ANOVA; * *p* < 0.05, ** *p* < 0.01, and n.s.: no significance.

To investigate whether leucine deprivation‐modified microbiota directly contribute to the metabolic homeostasis, we transplanted the fecal microbiota from mice fed with control diet (Cont‐FMT) or leucine‐deprived diet ((‐) Leu‐FMT) into the antibiotic pretreated high‐fat diet (HFD) mice (**Figure** **1**A; Figure S1A, Supporting Information). As expected, the amount of stool‐extracted DNA was 20‐fold lower after antibiotic treatment, compared to that before antibiotic treatment (Figure S1B, Supporting Information), suggesting antibiotic nearly depleted the inherent microbes. Next, we found that (‐) Leu‐FMT group showed improved insulin sensitivity, as demonstrated by decreased fed blood glucose levels (Figure 1B), decreased fed and fasting serum insulin levels (Figure 1C), decreased homeostasis model assessment of insulin resistance (HOMA‐IR) index (Figure 1D), and improved glucose tolerance test (GTT) (Figure 1E) and insulin tolerance test (ITT) (Figure 1F). Moreover, (‐) Leu‐FMT mice exhibited a tendency of decrease in body weight but markedly decrease in fat mass (Figure S1C, Supporting Information; Figure 1G). (‐) Leu‐FMT mice also showed decreased cell size of subcutaneous white adipose tissue (sWAT) (Figure 1H). Next, we investigated why the fat mass was decreased by (‐) Leu‐FMT. Our previous study found that (‐) Leu could induce sWAT browning,^[^ ^28^ ^]^ which contributed to fat loss. Therefore, we detected browning related genes (including uncoupling protein‐1 (*Ucp1*), peroxisome proliferator‐activated receptor gamma co‐activator 1α (*Pgc1α*), PR domain containing 16 (*Prdm16*), and cell death‐inducing DFFA‐like effector a (*Cidea*)) levels and UCP1 protein expression in the current study. Interestingly, browning related genes and UCP1 protein expression were all upregulated in the (‐) Leu‐FMT group, compared to that in the Cont‐FMT group (Figure 1I,J). However, the food intake, energy expenditure, and fecal energy loss had no significant changes between the two groups (Figure S1D–F, Supporting Information). These observations indicated that the gut microbiota modified by leucine deprivation was sufficient to bring metabolic benefits.

To further determine whether the gut microbiota is necessary for leucine deprivation induced metabolic improvements, we measured the effects of leucine deprivation in antibiotic‐treated mice (Figure 1K). Notably, leucine deprivation improved insulin sensitivity in non‐antibiotic mice, but these changes were abrogated by antibiotic treatment (Figure 1L–P). Although (‐) Leu had similar effects on reducing body weight and fat mass in both antibiotic and non‐antibiotic mice, the extent of fat mass decline in antibiotic mice was lower than that in the non‐antibiotic mice (Figure S1G,H, Supporting Information). Moreover, we found (‐) Leu induced decrease in sWAT cell size and increase in *Ucp1* gene and protein expression were reversed by antibiotic treatment (Figure S1I–K, Supporting Information). Other browning related genes increased by leucine deprivation were not affected by antibiotic treatment (Figure S1J, Supporting Information). These results indicated that (‐) Leu induced improvement in insulin sensitivity was dependent on microbiota. However, (‐) Leu induced fat loss and sWAT browning was partially mediated by microbiota.

### 2.2 Leucine Deprivation Alters Microbiota Composition and Increases *B. coccoides* Abundance

**Figure 2:**
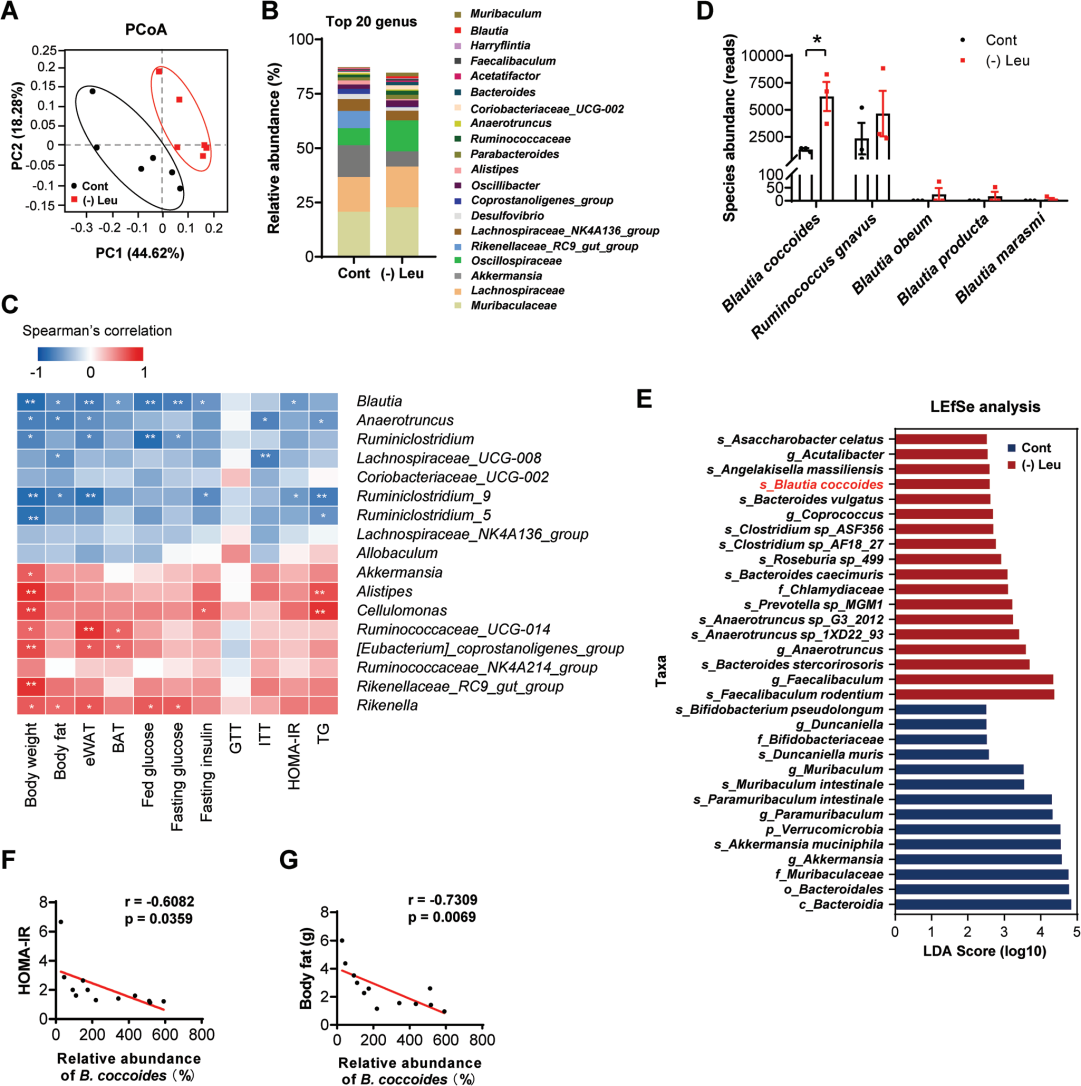
Leucine deprivation alters microbiota composition and increases *B. coccoides* abundance in mice. The cecal microbiota from 12‐week‐old male C57BL/6J WT mice treated with control diet (Cont) or leucine‐deprived diet ((‐) Leu) were used for bacterial 16S rDNA (A–C, F, and G, *n* = 6 biological replicates per group) and metagenomic sequencing (D, E, *n* = 3 biological replicates per group). A) PCoA of the gut microbiota structure based on Bray‐Curtis distance. B) Bacterial taxonomic profiling in the top 20 bacterial genus levels. C) Spearman's correlation analysis of 18 genus changed by (‐) Leu with metabolic parameters. D) The sequencing reads of bacterial species from genus *Blautia*. E) LEfSe analysis of metagenomic sequencing data. F) Spearman's correlation analysis of the relative abundance of *B. coccoides* with HOMA‐IR index. G) Spearman's correlation analysis of the relative abundance of *B. coccoides* with body fat. All values are expressed as the mean ± SEM. Statistical comparisons were carried out by nonparametric Mann‐Whitney *U* test; * *p* < 0.05, **p < 0.01.

We next performed 16S rDNA amplicon sequencing to investigate the alteration of gut microbiota composition induced by leucine deprivation. Principal‐coordinate analysis (PCoA) based on Bray‐Curtis distance indicated that leucine deprivation remarkably changed the gut microbiota structure (**Figure** **2**A), though the shannon index showed that alpha diversity had no change (Figure S2A, Supporting Information). Furthermore, many bacteria abundance were markedly altered at the phylum and genus levels (Figure 2B; Figure S2B, Supporting Information). These results demonstrated that the composition of the gut microbiota was rapidly and substantially altered in response to leucine deprivation.

We further explored the association between the selective microbiota and the metabolic parameters. Among the 18 significantly altered bacterial genera, we focused on *Blautia*, as they were significantly and negatively correlated with multiple metabolic parameters (including body weight, body fat, fed and fasting glucose levels, insulin levels, and HOMA‐IR index, etc) (Figure 2C). Consistently, studies from human trials revealed that genus *Blautia* was inversely associated with visceral fat accumulation.^[^ ^29^ ^]^ To further determine the alteration of the gut microbiota composition at species levels, we performed metagenomic sequencing. The results further revealed that the gut microbiota composition was substantially reshaped by leucine deprivation (Figure S2C,D, Supporting Information). Specially, we identified 5 bacterial species among the genus *Blautia*, but only *Blautia coccoides* was significantly increased by leucine deprivation (Figure 2D). *B. coccoides*, which belongs to the *Clostridia* class, is one of the major intestinal microbes in animals and humans.^[^ ^30^ ^]^ Furthermore, LEfSe analysis of metagenomic sequencing data illustrated that *B. coccoides* was enriched in leucine deprived mice (Figure 2E). Moreover, using Spearman's correlation analysis, we further confirmed that *B. coccoides* abundance was negatively correlated with both HOMA‐IR index and body fat (Figure 2F,G), suggesting that *B. coccoides* was beneficial for metabolic processes. We therefore investigated its biological characterization and effects on metabolic regulation.

For this study, we obtained the *B. coccoides* GA1 strain.^[^ ^31^ ^]^ Using scanning electron microscopy, we noted the coccoid or oval shape of *B. coccoides* GA1 strain, with a length of ≈0.4–0.8 µm and a width of ≈0.6–1.2 µm, and with monococcus or diplococcus form (Figure S2E, Supporting Information). We then performed whole‐genome sequencing of *B. coccoides* GA1 to predict its biological functions. The complete genome size of *B. coccoides* GA1 was ≈6.22 M nucleotides, which encoded 5845 genes (Figure S2F, Supporting Information). Phylogenetic tree analysis showed that *B. coccoides* GA1, *Blautia sp* YL58, and *Blautia_coccoides* were clustered on the same branch (Figure S2G, Supporting Information). Furthermore, the Kyoto Encyclopedia of Genes and Genomes pathway analysis showed that the encoded genes of *B. coccoides* GA1 genome mainly involved in membrane transport, signal transduction, and in carbohydrate, amino acid, and energy metabolisms (Figure S2H, Supporting Information).

### 2.3 *B. coccoides* Alleviates Metabolic Disorders in HFD and Gnotobiotic Mice

**Figure 3:**
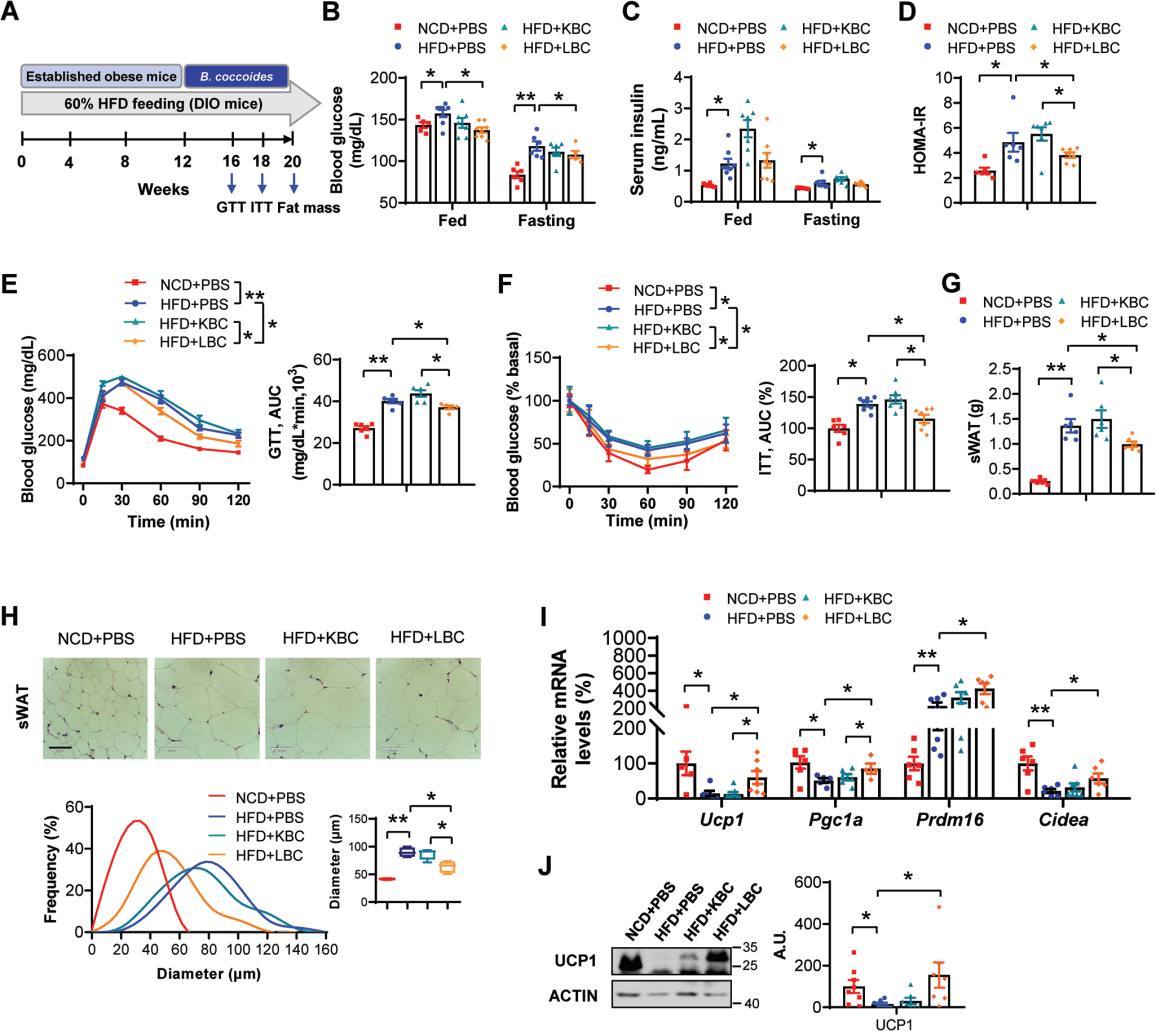
*B. coccoides* improves metabolic disorders in HFD mice. NCD and HFD mice were either orally gavage with PBS, heat‐killed *B. coccoides* GA1 (KBC), or live *B. coccoides* GA1 (LBC) for 8 weeks (*n* = 6–8 biological replicates per group). A) Experimental procedures. B) Fed and fasting blood glucose levels. C) Fed and fasting serum insulin levels assayed by ELISA. D) HOMA‐IR index. E) Glucose tolerance tests. The right panel is the AUC. F) Insulin tolerance tests (0.75 U kg^−1^). The right panel is the AUC. G) sWAT weight. H) The H&E staining of sWAT. Scale bars, 50 µm. The bottom panel is the frequency distribution of adipocyte cell size in sWAT and the box plot is average adipocyte diameter. I) Real‐time PCR analysis of browning related genes (*Ucp1*, *Pgc1α*, *Prdm16*, and *Cidea*) in sWAT. J) Western blot analysis of UCP1 protein levels in sWAT. The right panel is the densitometry analysis of UCP1 protein levels. A.U.: arbitrary units. All values are expressed as the mean ± SEM. Statistical comparisons were carried out by one‐way ANOVA; *p < 0.05 and **p < 0.01.

We next investigated whether *B. coccoides* could alleviate metabolic disorders. 4‐week‐old male wild type (WT) mice were fed with normal chow diet (NCD) or HFD for 12 weeks, and then they were administrated with either PBS, heat‐killed *B. coccoides* GA1 (KBC), or live *B. coccoides* GA1 (LBC) by oral gavage for additional 8 weeks (**Figure** **3**A). As expected, oral administration of LBC to HFD mice substantially enhanced the abundance of *B. coccoides* in the murine feces, compared to that in PBS treated HFD mice; however, the abundance of *B. coccoides* was not affected in KBC treated HFD mice (Figure S3A,B, Supporting Information). We further observed that LBC ameliorated HFD‐induced insulin resistance, as shown by reduced fed and fasting blood glucose levels, reduced HOMA‐IR index, and improved GTT and ITT, compared to that in PBS or KBC treated HFD mice (Figure 3B–F). In addition, we evidenced a considerable rescue effect on liver insulin signaling in LBC treated HFD mice, characterized by increased phosphorylation of insulin receptor (IR), protein kinase B (AKT), and glycogen synthase kinase 3β (GSK3β) (Figure S3C, Supporting Information). Furthermore, LBC did not exhibit a significant difference in food intake or body weight, whereas the sWAT weight and adipocyte size were decreased (Figure S3D,E, Supporting Information; Figure 3G,H). Browning related genes and UCP1 protein expression in the sWAT were increased in LBC treated HFD mice, compared to that in PBS or KBC treated HFD mice (Figure 3I,J). Given that strains from different origins of the same bacteria species may show different effects on host metabolic outcomes, we further confirm the metabolic improvements by gavage of HFD mice with *B. coccoides* DSM935 strain. Consistently, after 8 weeks of live *B. coccoides* DSM935 gavage, the HFD mice exhibited markedly improved insulin sensitivity, reduced fat mass, and increased sWAT browning (Figure S4A–K, Supporting Information). Nevertheless, no change in food intake, energy expenditure, and fecal energy loss was observed after *B. coccoides* DSM935 treatment (Figure S4L–N, Supporting Information). Taken together, these findings indicated that both the two *B. coccoides* strains improved metabolic disorders induced by HFD.

In order to confirm the direct effects of *B. coccoides* in metabolic regulation, the NCD‐fed non‐antibiotic or antibiotic treated mice were gavage with *B. coccoides* or PBS, respectively (Figure S5A–C, Supporting Information). Of interest, the antibiotic‐treated mice gavage with *B. coccoides* had markedly improved insulin sensitivity and a tendency of reducing fat mass (Figure S5D–I, Supporting Information). Moreover, these effects were also found in antibiotic‐treated HFD mice that were orally gavage with *B. coccoides* (Figure S5J–P, Supporting Information). Collectively, these findings demonstrated that singly colonization of *B. coccoides* into gnotobiotic mice had significant beneficial effects on improving metabolic disorders.

### 2.4 *B. coccoides* Increases the Circulating I3AA Levels via Metabolizing Tryptophan

**Figure 4:**
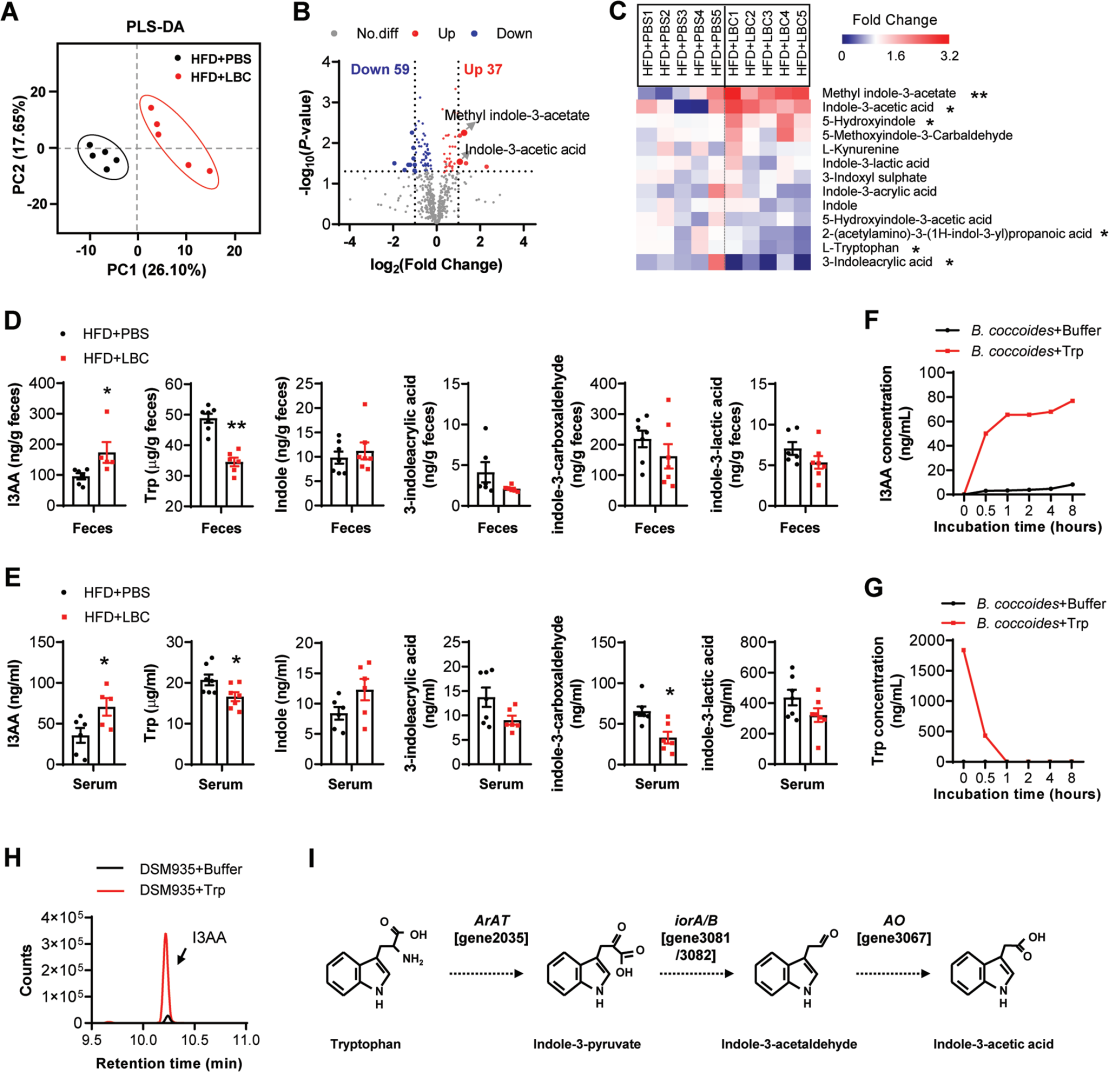
*B. coccoides* increases the circulating I3AA via metabolizing tryptophan. A–E) HFD mice were either orally gavage with PBS or live *B. coccoides* (LBC) for 4 weeks. Serum was collected for untargeted metabolomics profiling (*n* = 5–7 biological replicates per group). A) Partial least squares discriminant analysis (PLS‐DA) of metabolite composition. B) Volcano chart of detected metabolites. The indicate plot was methyl indole‐3‐acetate and I3AA. C) Heat map of tryptophan metabolites. D,E) The levels of I3AA, tryptophan (Trp), and its indole metabolites in cecal feces (D) and sera (E) detected by LC‐MS/MS analysis, respectively. F,G) Incubated the *B. coccoides* GA1 strain with Trp for the indicated time. I3AA (F) and Trp (G) concentrations in the supernatants were detected by LC‐MS/MS analysis, respectively. H) Incubated the *B. coccoides* DSM935 strain with Trp for 8 h. I3AA levels in the supernatants were detected by LC‐MS/MS. I) The probable metabolic pathway for generating I3AA. *ArAT*, aromatic amino acid aminotransferase; *iorA/B*, Indolepyruvate ferredoxin oxidoreductase subunit alpha/beta; *AO*, aldehyde oxidase. All values are expressed as the mean ± SEM. Statistical comparisons were carried out by unpaired two‐tailed Student's *t*‐test; *p < 0.05 and **p < 0.01.

Numerous studies indicated that metabolites produced by gut microbiota contributed to host metabolism.^[^ ^32^ ^]^ By adding different proportions of *B. coccoides* cultural supernatants into the primary hepatocytes medium, the insulin signaling pathway was significantly activated (Figure S6A, Supporting Information), suggesting metabolites might mediate the beneficial effects of *B. coccoides*. To investigate the association between *B. coccoides*‐derived metabolites and host metabolism, we performed untargeted metabolomics profiling of the sera derived from LBC or PBS treated HFD mice. A total of 577 metabolites, including lipids, amino acids, carbohydrates, and bile acids were detected. The plot of the partial least squares‐discriminant analysis revealed a substantially altered metabolites composition after LBC treatment (**Figure** **4**A). We identified 96 metabolites that differed between LBC and PBS treated HFD mice. Among them, 37 were increased and 59 were significantly reduced (Figure 4B; Table S1, Supporting Information). These metabolites showed marked enrichment in certain biological processes: beta‐alanine and tryptophan metabolisms, malate‐aspartate shuttle, and beta‐oxidation of very long‐chain fatty acids (Figure S6B, Supporting Information).

Subsequently, we particularly focused on tryptophan metabolites, because many studies indicated that some indole derivatives produced from tryptophan metabolism by intestinal microorganisms acted as aryl hydrocarbon receptor (AhR) agonists to promote metabolic benefits.^[^ ^33^, ^34^ ^]^ Interestingly, the levels of both methyl indole‐3‐acetate and I3AA increased over 2 folds, whereas tryptophan levels were significantly decreased in murine sera of the LBC group, compared to that in the PBS group (Figure 4B,C). However, the levels of other tryptophan metabolites, such as 5‐methoxyindole‐3‐ carbaldehyde, indole‐3‐lactic acid, 3‐Indoxyl sulphate, indole‐3‐acrylic acid, and indole had no change between the two groups (Figure 4C). We further quantified tryptophan and its metabolites levels in the murine cecal feces, sera, and tissues using liquid chromatography‐tandem mass spectrometry (LC‐MS/MS). The levels of I3AA were predominantly elevated after LBC treatment in the cecal feces, sera, liver, and sWAT, compared to that in PBS group (Figure 4D,E; Figure S6C,D, Supporting Information). Meanwhile, the tryptophan levels were significantly decreased in both cecal feces and sera of LBC mice (Figure 4D,E). Other tryptophan metabolites were not affected by LBC (Figure 4D,E). The AhR activity in the liver and sWAT was increased, marked by an upregulated expression of its targeted genes cytochrome P450 family 1 subfamily A member 1 (*Cyp1a1*) or cytochrome P450 family 1 subfamily B member 1 (*Cyp1b1*) (Figure S6E,F, Supporting Information). Consistently, both the feces and serum I3AA levels were markedly reduced in HFD mice compared to that in NCD mice, whereas the serum I3AA levels were significantly increased in (‐) Leu mice, as compared to that in Cont mice (Figure S6G–I, Supporting Information). These results suggested that I3AA produced by (‐) Leu‐*B. coccoides* axis might be involved in metabolic improvements.

To further evaluate whether *B. coccoides* could directly metabolize tryptophan into I3AA, we cultured the *B. coccoides* strains in a defined buffer containing tryptophan at appropriate concentrations. At different incubation times, we assessed the I3AA and tryptophan concentrations in the supernatants. The results showed that under *B. coccoides* GA1 incubation, I3AA was robustly accumulated after 0.5 h in the supernatants added with tryptophan, compared to that in the vehicle control (Figure 4F). Consistently, tryptophan was quickly consumed by *B. coccoides* GA1 (Figure 4G). Moreover, the ability of metabolizing tryptophan to produce I3AA was also confirmed by *B. coccoides* DSM935 strain (Figure 4H). We found multiple I3AA‐producing metabolic enzyme genes, including the rate‐limiting enzyme aromatic amino acid aminotransferase,^[^ ^35^ ^]^ indole pyruvate ferredoxin oxidoreductase A/B, and aldehyde oxidase in the genome of *B. coccoides* (Figure 4I; Table S2, Supporting Information). These findings suggested that *B. coccoides* increased I3AA levels via metabolizing tryptophan.

### 2.5 I3AA has Beneficial Effects on Metabolic Improvements

**Figure 5:**
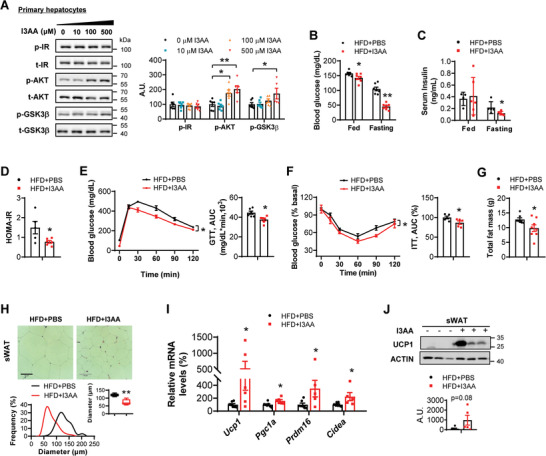
I3AA has beneficial effects on metabolic improvements in vitro and in vivo. A) Primary hepatocytes were incubated with indicate dose of I3AA for 48 h and then stimulated with 100 nM insulin for 20 min (*n* = 6 replicates per group). Western blot analysis of p‐IR, p‐AKT, and p‐GSK3β levels. The right panel is the densitometry analysis of the relative abundance of phosphorylated proteins normalized to their total protein levels. A.U.: arbitrary units. B–J) HFD mice were orally gavage with PBS or 10 mg kg^−1^ I3AA for 4 weeks (*n* = 6–7 biological replicates per group). B) Fed and fasting blood glucose levels. C) Fed and fasting serum insulin levels assayed by ELISA. D) HOMA‐IR index. E) Glucose tolerance tests. The right panel is the AUC. F) Insulin tolerance tests (0.5 U kg^−1^). The right panel is the AUC. G) Total fat mass. H) The H&E staining of sWAT. Scale bars, 50 µm. The bottom panel is the frequency distribution of adipocyte cell size in sWAT and the box plot is average adipocyte diameter. I) Real‐time PCR analysis of browning related genes (*Ucp1*, *Pgc1α*, *Prdm16*, and *Cidea*) in sWAT. J) Western blot analysis of UCP1 protein levels. The bottom panel is the densitometry analysis of UCP1 protein levels. All values are expressed as the mean ± SEM. Statistical comparisons were carried out by unpaired two‐tailed Student's *t*‐test; *p < 0.05 and **p < 0.01.

A previous study indicated that I3AA repressed liver lipogenesis by negatively regulating both fatty acid synthase and sterol regulatory element‐binding protein‐1c gene expression;^[^ ^33^ ^]^ however, the roles of I3AA in regulating liver insulin sensitivity and sWAT browning remain unclear. We confirmed that primary hepatocytes cultured with either 100 or 500 µM I3AA for 48 h significantly stimulated insulin signaling (**Figure** **5**A; Figure S7A, Supporting Information). However, I3AA treatment did not affect browning related genes expression in differentiated adipocytes (Figure S7B, Supporting Information), indicating that I3AA had no direct effect on white adipocytes browning.

To further analyze whether I3AA could promote metabolic benefits in vivo, we first tested the effective dose of I3AA on activating liver AhR activity. Finally, we chose 10 mg kg^−1^ I3AA in the current study (Figure S7C, Supporting Information). HFD mice were either orally gavage with PBS or 10 mg kg^−1^ I3AA for 4 weeks (Figure S7D, Supporting Information). We found that serum I3AA levels were significantly increased after I3AA supplementation (Figure S7E, Supporting Information). Oral administration of I3AA improved insulin sensitivity, reduced fat mass, and induced sWAT browning in HFD mice, compared to that in PBS treated HFD mice (Figure 5B–J). Although the body weight, food intake, energy expenditure, and fecal energy loss had no difference between the two groups (Figure S7F–I, Supporting Information).

### 2.6 Liver AhR is Essential for *B. coccoides*‐I3AA Axis Induced Metabolic Improvements

Studies in animal models suggested that AhR played a significant role in regulating metabolism^[^ ^34^, ^36^ ^]^ and I3AA was a ligand of AhR.^[^ ^33^ ^]^ Notably, several publications revealed that activation of AhR in adipose tissue contributed to diet‐induced obesity.^[^ ^37^, ^38^ ^]^ In addition, adipose‐specific AhR depletion protected from diet induced obesity.^[^ ^39^ ^]^ These reports suggested that *B. coccoides*‐I3AA induced fat loss was probably not depend on activating adipose tissue AhR directly. Also considering the results in Figure S7B, Supporting Information, we focused on liver AhR signaling in regulating metabolic homeostasis.

To evaluate the role of liver AhR in regulating metabolic homeostasis, we first conducted in vitro experiments. Primary hepatocytes were transfected with siRNA to block *AhR* expression, prior to I3AA treatment. Knockdown of *AhR* abrogated I3AA stimulated activation of AhR and insulin signaling (Figure S8, Supporting Information). These results suggested that I3AA activated insulin signaling was dependent on hepatic AhR.

**Figure 6:**
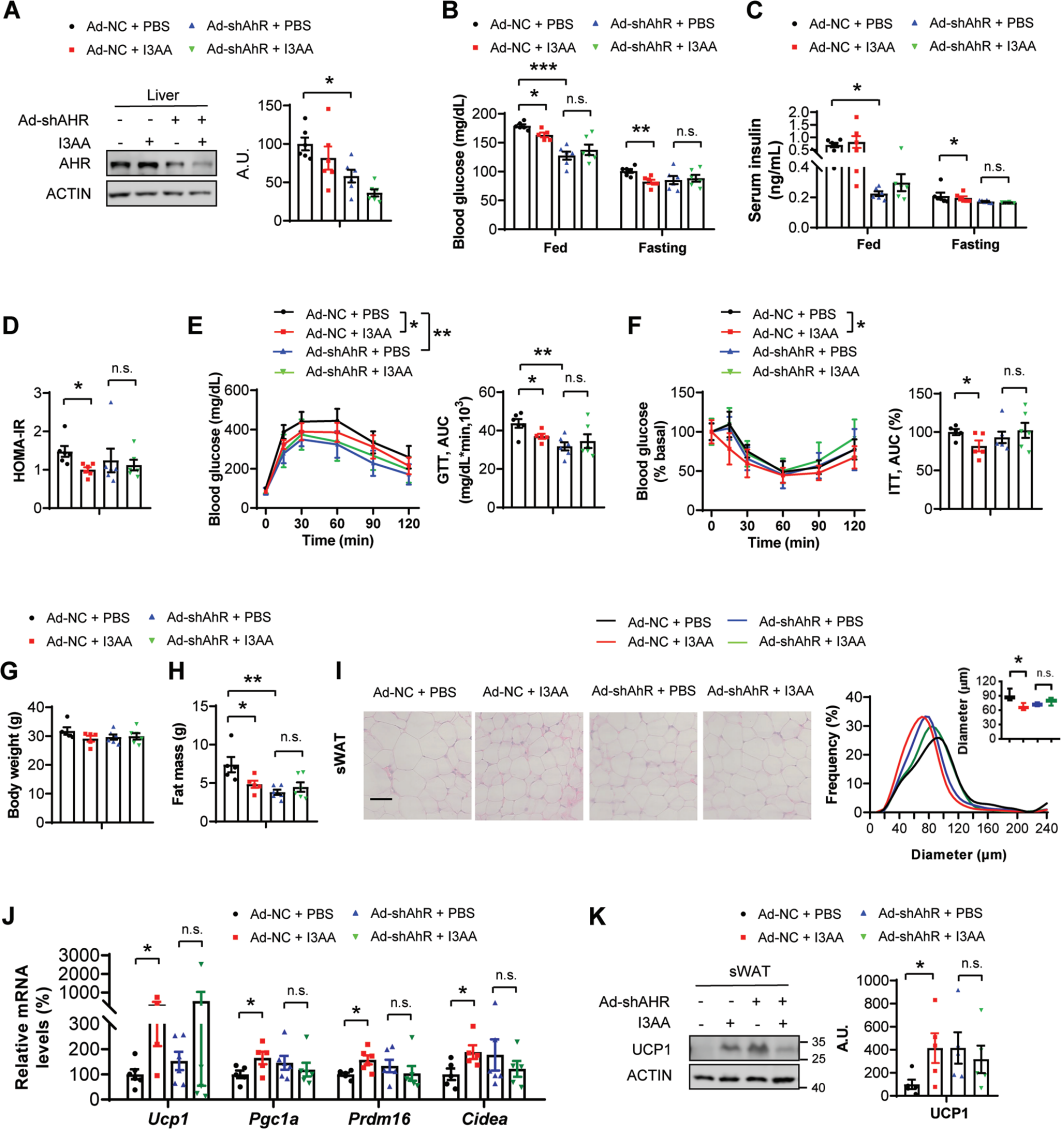
Liver AhR is essential for I3AA induced metabolic improvements. HFD mice were injected with control adenovirus (Ad‐NC) or adenovirus expressing small‐hairpin RNA specific for mouse *AhR* (Ad‐sh*AhR*) via the tail vein, respectively. The Ad‐NC or Ad‐sh*AhR* mice were either orally gavage with PBS or 10 mg kg^−1^ I3AA daily for 10 days (*n* = 5–6 biological replicates per group). A) Western blot analysis of AhR protein levels in the liver. The right panel is the densitometry analysis of AhR protein levels. A.U.: arbitrary units. B) Fed and fasting blood glucose levels. C) Fed and fasting serum insulin levels assayed by ELISA. D) HOMA‐IR index. E) Glucose tolerance tests. The right panel is the AUC. F) Insulin tolerance tests (0.5 U kg^−1^). The right panel is the AUC. G) Body weight. H) Total fat mass. I) The H&E staining of sWAT. Scale bars, 50 µm. The right panel is the frequency distribution of adipocyte cell size in sWAT and the box plot is average adipocyte diameter. J) Real‐time PCR analysis of the gene expression related to sWAT browning, including *Ucp1*, *Pgc1α*, *Prdm16*, and *Cidea*. K) Western blot analysis of UCP1 protein levels. The right panel is the densitometry analysis of UCP1 protein levels. All values are expressed as the mean ± SEM. Statistical comparisons were carried out by two‐way ANOVA; *p < 0.05, **p < 0.01, ***p < 0.001, and n.s.: no significance.

For in vivo studies, 4‐week‐old male WT mice were fed with HFD for 2 months, and then they were injected with negative control adenovirus (Ad‐NC) or adenovirus expressing small‐hairpin RNA specific for mouse *AhR* (Ad‐*shAhR*) via tail vein, respectively. The Ad‐NC or Ad‐*shAhR* mice were either orally gavage with PBS or 10 mg kg^−1^ I3AA daily for 10 days. First, AhR expression was knocked down in the livers of mice injected with Ad‐*shAhR*, compared to that in Ad‐NC mice (**Figure** **6**A). I3AA induced improvements in insulin sensitivity were reversed by knocking down liver AhR (Figure 6B–F). To our surprise, although there was no change in body weight, I3AA induced fat loss, decreased sWAT cell size, increased browning genes, and upregulated UCP1 protein expression were all reversed by liver specific AhR knockdown (Figure 6G–K). These results demonstrated that I3AA induced improvements in insulin sensitivity, fat loss, and sWAT browning were dependent on liver AhR.

We also confirmed whether AhR mediated *B. coccoides*‐induced metabolic improvements using *AhR* knockout (*Ahr* ^−/−^) mice (Figure S9A,B, Supporting Information). *Ahr* ^−/−^ mice had no remarkable metabolic phenotypes when fed on NCD (Figure S9C–E, Supporting Information). Notably, we found that glucose and lipid related metabolic parameters improved by *B. coccoides* were markedly abrogated in HFD‐fed *Ahr* ^−/−^ mice (Figure S9F–N, Supporting Information).

We further explored whether *B. coccoides* induced metabolic improvements were dependent on liver AhR. HFD mice were injected with negative control adeno‐associated virus (AAV‐NC) or adeno‐associated virus expressing small‐hairpin RNA specific for mouse *AhR* (AAV‐*shAhR*) via tail vein, respectively. 1 month later, both groups were gavage with LBC for 4 weeks. We also confirmed that under *B. coccoides* treatment conditions, the insulin sensitivity and liver insulin signaling were all inhibited in AAV‐*shAhR* group, compared to that in the AAV‐NC group (Figure S10A–G, Supporting Information). However, there was no change in fat mass between AAV‐sh*AhR* and AAV‐NC mice under *B. coccoides* treatment, which was possible that the *B. coccoides* treatment time was not long enough (Figure S10H,I, Supporting Information). These results indicated that *B. coccoides* induced improvement in insulin sensitivity was partially relied on liver AhR.

Finally, we wondered if leucine deprivation could also improve metabolic homeostasis when knocking down liver AhR. 12‐week‐old male WT mice were injected with Ad‐NC or Ad‐*shAhR* via tail vein, respectively. After 3 days, Ad‐NC and Ad‐*shAhR* mice were either fed with Cont diet or (‐) Leu diet for another 7 days. We found that although (‐) Leu diet induced decrease in fed insulin levels and ITT were not affected by knocking down liver AhR, (‐) Leu diet induced improvements in other glucose metabolism parameters, like fed and fasting blood glucose levels, HOMA‐IR index, as well as GTT, were reversed by knocking down liver AhR (Figure S11A–F, Supporting Information). Moreover, (‐) Leu diet induced fat loss, decreased adipocyte size, increased browning genes, and upregulated UCP1 protein levels were all reversed by liver specific AhR knockdown (Figure S11G–K, Supporting Information). These results demonstrated that (‐) Leu induced metabolic improvements were partially dependent on liver AhR.

However, the mechanisms underlying improved insulin sensitivity and sWAT browning induced by I3AA‐liver AhR are still unclear. Hepatokine fibroblast growth factor‐21 (FGF21), which is mainly expressed in the liver, is a novel target gene of AhR.^[^ ^40^, ^41^ ^]^ Importantly, FGF21 plays important roles in regulating liver insulin sensitivity and white adipose tissue browning.^[^ ^42^, ^43^ ^]^ Therefore, we speculated that *B. coccoides*‐I3AA‐liver AhR axis might regulate glucose and lipid homeostasis via hepatokine FGF21. As we expected, liver and serum FGF21 levels were all upregulated in mice fed with (‐) Leu diet for 7 days (Figure S12A, Supporting Information). Similarly, liver and serum FGF21 levels were all upregulated in HFD mice treated with *B. coccoides* DSM 935 strain or I3AA, respectively (Figure S12B,C, Supporting Information). Importantly, I3AA induced increase in liver and serum FGF21 levels were blocked by Ad‐*shAhR* (Figure S12D, Supporting Information). Therefore, FGF21 might mediate the effect of *B. coccoides*‐I3AA‐liver AhR axis in regulating metabolic homeostasis. However, the participant of FGF21 in *B. coccoides*‐I3AA‐liver AhR induced metabolic improvements need further investigation.

Overall, (‐) Leu‐*B. coccoides*‐I3AA axis activated liver AhR and contributed to the improvement of metabolic disorders.

### 2.7 Fecal *B. coccoides* and I3AA Levels are Correlated with Human Metabolic Syndrome

**Figure 7:**
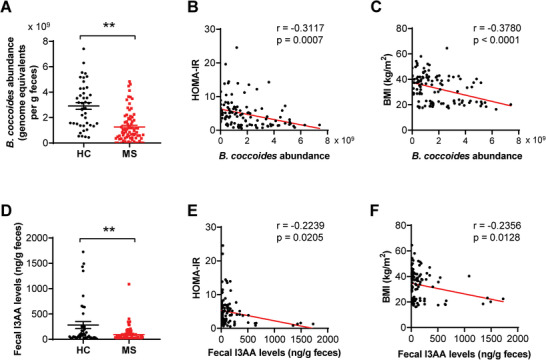
Fecal *B. coccoides* and I3AA levels are correlated with human metabolic syndrome. A) The absolute abundance of *B. coccoides* in feces from subjects with metabolic syndrome (MS; *n* = 78) and healthy controls (HC; *n* = 43). B,C) Spearman's correlation analysis of the *B. coccoides* abundance with HOMA‐IR (B) or BMI (C), respectively. D) The concentrations of fecal I3AA in MS (*n* = 78) and health individuals (*n* = 43). E,F) Spearman's correlation analysis of the fecal I3AA levels with HOMA‐IR (E) or BMI (F), respectively. Statistical comparisons were carried out by the nonparametric Mann–Whitney *U* test; **p < 0.01.

Metabolic syndrome is a common metabolic disorder that results from the increasing prevalence of obesity, which increases the risk of type 2 diabetes and cardiovascular diseases.^[^ ^44^ ^]^ We further explored whether the alteration of *B. coccoides*‐I3AA axis was relevant to human disease by analyzing fecal samples from healthy subjects and individuals with metabolic syndrome. Compared with the healthy individuals, the individuals with metabolic syndrome had lower abundance of *B. coccoides* (**Figure** **7**A). Furthermore, the abundance of *B. coccoides* showed significantly negative correlation with HOMA‐IR and body mass index (BMI) (Figure 7B,C). Consistently, fecal I3AA levels from individuals with metabolic syndrome were significantly lower than that in healthy subjects (Figure 7D). We also observed that fecal I3AA levels had negative correlation with HOMA‐IR and BMI, respectively (Figure 7E,F). Collectively, these clinical data demonstrated a decrease of *B. coccoides* and I3AA levels in metabolic syndrome, which also suggested the potential role of *B. coccoides*‐I3AA in the pathogenesis of metabolic syndrome.

**Figure 8:**
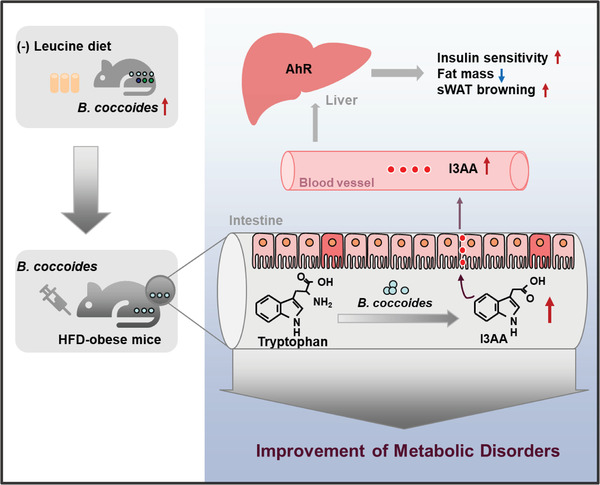
Graphic abstract of the function and underlying mechanism of *B. coccoides* in ameliorating metabolic disorders. Leucine deprivation increases the abundance of *B. coccoides*, which improves insulin sensitivity, induces sWAT browning, and reduces body fat in HFD mice by metabolizing tryptophan into I3AA that activates AhR in the liver.

Overall, we found that leucine deprivation increased the abundance of *B. coccoides*, which improved insulin sensitivity and reduced body fat in HFD mice by metabolizing tryptophan into I3AA that activated AhR in the liver. Our findings provide new insights for improving metabolic disorders (**Figure** **8**).

## 3 Discussion

Gut microbiota plays a pivotal role in host health and its composition can be modulated by nutrition, which has been connected to aspects of human metabolic diseases.^[^ ^45^ ^]^ Here, we found that transplanting the microbiota from leucine deprived mice into gnotobiotic mice improved metabolic dysfunction, suggesting that some intestinal bacteria modulated by leucine deprivation may play key roles in metabolic regulation. In fact, with an increasing understanding of the complicated interaction between the gut microbiota and host, studies on bacterial species or strain levels are urgently required to define the exact functions of specific bacteria on causing disease or maintaining health. Notably, through 16S rDNA and metagenomic sequencing, we confirmed that the composition of gut microbiota was rapidly and substantially altered in response to leucine deprivation, especially the significant increase of *B. coccoides* levels. *B. coccoides* was linked to certain diseases, such as type I diabetes^[^ ^46^ ^]^ and irritable bowel syndrome.^[^ ^47^ ^]^ In this study, we demonstrated that *B. coccoides* was effective in improving metabolic dysfunctions, which made *B. coccoides* an important contributing factor in the maintenance of metabolic homeostasis. Our work may constitute an intervention strategy for treating metabolic syndrome.

Metabolites derived from the gut microbiota, such as short chain fatty acids, bile acid, and amino acid derivatives, serve as important signals for the crosstalk between gut microbiota and host target tissues. These metabolites contribute to the appropriate regulation of the host physiology and metabolism.^[^ ^10^, ^11^, ^48^ ^]^ For example, *Prevotella copri* and *Bacteroides vulgatus* are the main species driving the association between BCAA biosynthesis and insulin resistance.^[^ ^49^ ^]^ As such, defining and characterizing different microbiota‐derived metabolites could help to clarify the characterization of gut microbiota composition and function, to mechanistically understand the host‐microbiota crosstalk, and to identify possible biomarkers or signatures of health and disease. To explore the underlying mechanisms of *B. coccoides* in improving metabolic disorders, we investigated the metabolites altered by *B. coccoides*. In this study, both the cecal feces and serum I3AA levels were markedly increased, whereas tryptophan was significantly decreased, in HFD mice gavage with *B. coccoides*. Tryptophan is an essential amino acid for humans, and its metabolism in the gut generates several molecules such as indole and indole derivatives. Many indole derivatives, such as indole‐3‐aldehyde, I3AA, indole‐3‐propionic acid, indole‐3‐acetaldehyde, and indoleacrylic acid, are ligands for AhR.^[^ ^50^ ^]^ In fact, studies have reported that AhR has protective effects against diet‐induced metabolic disorders.^[^ ^51^ ^]^ The impairment of gut microbiota to produce tryptophan‐based AhR ligands is associated with the pathogenesis of metabolic syndrome.^[^ ^33^, ^34^ ^]^ Importantly, a previous study reported lower concentrations of I3AA in fecal samples from obese individuals or mice, compared to those from non‐obese individuals or mice, respectively.^[^ ^34^ ^]^ Therefore, we hypothesized whether *B. coccoides* could directly produce I3AA that mediated the beneficial effects. To date, only a few commensal species were reported to produce I3AA, such as *Clostridium bartlettii* ^[^ ^52^ ^]^ and *Bacteroides spp*.^[^ ^53^ ^]^ Notably, we discovered that incubation of *B. coccoides* with tryptophan directly produced I3AA and found multiple I3AA‐producing metabolic enzyme genes in the genome of *B. coccoides*; however, the metabolic pathways responsible to produce I3AA remain to be validated. Interestingly, a recent study indicated that microbiota‐derived I3AA increased the chemotherapy efficacy in pancreatic cancer;^[^ ^54^ ^]^ our study revealed the key role of *B. coccoides*‐I3AA on improving metabolic dysfunction. In addition, several works have reported that I3AA‐AhR axis could ameliorate non‐alcoholic fatty liver disease by negatively regulates several lipogenesis enzymes expression.^[^ ^33^ ^]^ Therefore, we could not exclude the possibility that improved hepatic insulin sensitivity was profited from *B. coccoides*‐I3AA‐AhR axis induced decreased fatty liver.

Furthermore, a study reported that colonization of a single *B. coccoides* drastically altered the metabolic environment and reshaped the *Escherichia coli* evolution in the mouse gut.^[^ ^55^ ^]^ Consistently, our study indicated that 4 weeks of *B. coccoides* treatment reshaped the gut microbiota profile in HFD mice (Figure S13, Supporting Information). Based on this result, we wondered the gut microbiota reshaped by *B. coccoides* was either the cause or the consequence of metabolic improvements. Previous studies reported that HFD mice had reduced number of observed species and alpha diversity, particularly the ratio of Bacteroidetes/Firmicutes;^[^ ^15^, ^16^ ^]^ however, these changes were rescued by gavage with *B. coccoides*. Nevertheless, we found that singly colonization of *B. coccoides* into gnotobiotic mice had significant beneficial effect on insulin sensitivity, suggesting that the improvements of insulin sensitivity induced by *B. coccoides* may independent on reshaping the gut microbiota.

Interestingly, we indicated that *B. coccoides* metabolized tryptophan into I3AA, which improved host metabolic dysfunction; however, the roles of *B. coccoides* on other aspects, such as depression or anxiety, induced by tryptophan‐metabolic disorders remain unclear.

## 4 Experimental Section

### Animal and Treatments

C57BL/6J WT mice were purchased from Shanghai Laboratory Animal Co., Ltd. (Shanghai, China). *Ahr* ^−/−^ mice were generated previously.^[^ ^56^ ^]^ *Ahr* ^−/−^ mice and littermate WT mice were kept separately in different cages after weaning at 4‐week‐old. Mice were housed in laboratory cages at 23 ± 1 °C, with 50 ± 10% relative humidity under a 12 h dark/light cycle and with free access to water and food. All mice were maintained under specific pathogen‐free conditions and animal research was complied with all relevant ethnic regulations. For all experiments, mice were sorted into multiple groups based on their body weight and fat mass. All animal experiments were performed in accordance with relevant guidelines and regulations approved by the Institutional Animal Care and Use Committee of Fudan University (Shanghai, China; No. 202203006S).

Control diet (Cont; nutritionally complete amino acid) and (‐) Leu diet were purchased from Research Diets, Inc. (New Brunswick, NJ, USA). All diets were isocaloric and with the same carbohydrate and lipid component. At the start of the feeding experiments, mice were acclimated to Cont diet for 3 days and then randomly divided into Cont and (‐) Leu groups, with free access to diets for 7 days, respectively. The diet details are described in Table S3, Supporting Information. Generally, for HFD‐related experiments, 4‐week‐old C57BL/6J male WT mice were pre‐fed with HFD (60% fat, Research Diet, D12492) for 12 weeks to induce metabolic disorders.

For liver specific knockdown of *AhR*, mice were injected with AAV‐sh*AhR* or Ad‐sh*AhR* via the tail vein, respectively. Total AAV volume was 200 µL and total virus titer was 1 × 10^11^ viral genomes per mice; the adenovirus volume was 200 µL and total virus titer was 1 × 10^9^ PFU per mice. The small‐hairpin RNA specific for mouse *AhR* was as follows: 5′‐GAGGGAUUAACUUCUAGAU‐3′.^[^ ^57^ ^]^

For the I3AA efficiency assay, HFD mice were orally gavage with 10 mg kg^−1^ I3AA (I5148, Sigma) or equivalent volume of sterile PBS daily for 30 days, respectively.

After experimental procedure, animals were euthanized by cervical dislocation, and appropriate tissues were harvested for further analysis.

### Human Subjects

Individuals with metabolic syndrome and healthy controls were recruited in the Department of Metabolic and Bariatric Surgery, First Affiliated Hospital of Jinan University (Guangzhou, China) and provided informed consent. None of the subjects had received antibiotics 3 months before collecting samples. Approval for human studies was obtained from the local ethics committee (registration number: KY‐2022‐009). Clinical characteristics and metabolic biomarkers that showed significant differences in the metabolic syndrome versus the healthy controls are summarized in Table S4, Supporting information.

### Cell Treatments

Mouse primary hepatocytes were prepared by collagenase perfusion as described previously.^[^ ^58^ ^]^ Cells were cultured in DMEM (Invitrogen, Carlsbad, CA, USA) with 10% fetal bovine serum, 100 units mL^−1^ penicillin, and 100 µg mL^−1^ streptomycin sulfates. No mycoplasma was detected in all cells that were used. To detect insulin signaling, primary hepatocytes was incubated with 100 nM insulin for 20 min before harvested. C3H10T1/2 (ATCC, CCL‐226) cell line was purchased from Cell Bank of Shanghai Institute of Cell Biology, Chinese Academy of Sciences. The differentiation of C3H10T1/2 into mature adipocytes was induced as previously described.^[^ ^59^ ^]^ For *AhR* knockdown, the siRNAs sequences were: 5′‐GAGGGAUUAACUUCUAGAU‐ 3′.^[^ ^57^ ^]^ Cells were transfected with siRNA using lipofectamine 3000 Transfection Reagent (invitrogen).

### Bacteria Strains, Culture, and Preparation

*B. coccoides* DSM935 (ATCC 29 236) strain was purchased from the German Collection of Microorganisms and Cell Cultures. *B. coccoides* GA1 strain obtained from Dr. Li's laboratory,^[^ ^31^ ^]^ which was identified by comparing the 16S rRNA gene sequences with the NCBI reference database (https://www.ncbi.nlm.nih.gov/). The *B. coccoides* GA1 strain showed 99.65% similarity to that of *B. coccoides* DSM 29 138 in GenBank with the accession number KU196081.1. The *B. coccoides* strains were routinely cultured in anaerobically sterilized ATCC 2722 medium (containing ground tryptic soy broth supplemented with Yeast extract, L‐Cysteine·HCL, Hemin and Vitamin K1) at 37 °C in an anaerobic chamber, with an atmosphere of 10% H_2_, 10% CO_2_, and 80% N_2_. All manipulations with *B. coccoides* were performed in the anaerobic chamber, with medium and reagents pre‐reduced for at least 48 h.

*B. coccoides* was 1:100 diluted and cultured for about 36 h. Cell pellets were obtained by centrifuging at 8000 × g for 10 min at 4 °C and then were washed twice with sterile anaerobic PBS. In the end, cell pellets were re‐suspended in sterile anaerobic PBS containing 20% v/v glycerol‐PBS to 5 × 10^10^ CFU mL^−1^ and sealed in 12 × 32 mm glass crimp top vials (200 µL per vial) to ensure anoxic conditions during long‐term −80 °C storage. In addition, *B. coccoides* was heat‐killed by autoclaving at 121 °C for 30 min. A viability confirmation was performed by culture showing that heat‐killed *B. coccoides* did not grow at all, while live *B. coccoides* grew well. Before administration to mice, live *B. coccoides* or heat‐killed *B. coccoides* stock were thawed and diluted 10 times. Mice were orally gavage daily with 200 µL 2%‐glycerol PBS, heat‐killed *B. coccoides*, or 1 × 10^9^ CFU live *B. coccoides* for 8 weeks, respectively.

### Antibiotic Treatments

For depletion of inherent microbes, fresh antibiotics were administered daily in the drinking water as described previously.^[^ ^60^ ^]^ Briefly, mice were provided with free access to autoclaved water containing 1 g L^−1^ ampicillin, 1 g L^−1^ neomycin, 0.5 g L^−1^ vancomycin, and 0.5 g L^−1^ meropenem for 4 weeks. Control mice were provided with autoclaved water. To verify the efficacy of the microbiota depletion, fecal pellets were collected for total DNA extraction and the content of bacterial 16S rRNA was analyzed by RT‐qPCR (forward primer: 5′‐TCCTACGGGAGGCAGCAGT‐3′; reward primer: 5′‐GGACTACCAGGGTATCTAATCCTGTT‐3′).

### Fecal Microbial Transplantation

Fecal microbial transplantation was performed based on an established protocol.^[^ ^61^ ^]^ Briefly, 10‐week‐old male donor mice (*n* = 10 per group) were fed with Cont or (‐) Leu diet for 7 days, and then euthanized by cervical dislocation and immediately transferred to an anaerobic chamber for next processes. The cecal and colonic feces samples from donor mice of each group were pooled, and homogenized in sterile anaerobic PBS by vigorously mixed for 3 min using a benchtop vortex. The homogenates were centrifuged at 800 g, 4 °C for 5 min to remove the impurity, and then the upper turbid liquids containing live microbes were filtered using a 70‐µm pore‐size nylon filter to remove the small particles and fibrous matter. The filtrates were centrifuged at 8000 g, 4 °C for 10 min to collect the bacterial particles. Subsequently, sterile anaerobic 2% glycerol‐PBS (1 mL/100 mg feces) was used to re‐suspend the precipitates and sealed in 12 × 32 mm glass crimp top vials to ensure anoxic conditions during long‐term −80 °C storage. Antibiotic treated HFD recipients were inoculated daily with fecal microbial (200 µL for each mouse) by oral gavage for 30 days.^[^ ^62^, ^63^ ^]^

### Metabolic Parameters, Glucose Tolerance Tests, Insulin Tolerance Tests, and HOMA‐IR Index

The body fat composition was determined using the Bruker Minispec mq10 NMR Analyzer (Bruker, Billerica, MA). Serum triglyceride (TG) levels were determined enzymatically using TG reagent (Wako, Japan). Levels of blood glucose and serum insulin were measured with a Glucometer Elite monitor or Mercodia Ultrasensitive Rat Insulin ELISA kit (ALPCO Diagnostics, Salem, NH), respectively. GTT was performed by intraperitoneal injection with 2 g kg^−1^ glucose after overnight fasting; ITT was performed by intraperitoneal injection with 0.5, 0.75, or 1 U kg^−1^ insulin after 4 h fasting as indicated. The HOMA‐IR index was calculated according to the following formula: (fasting glucose levels [mmol L^−1^]) × (fasting serum insulin [ng mL^−1^])/22.5.

### H&E Staining and Adipocyte Size Quantification

For standard histology, sections of sWAT were fixed overnight in 4% paraformaldehyde, embedded in paraffin, sectioned (5‐µm), and stained with hematoxylin and eosin. Adipocyte cell diameter in the H&E‐stained sections was measured using Image J with over 150–200 adipocytes per mouse and ≈3–5 mice per group.^[^ ^64^ ^]^

### Scanning Electron Microscope

*B. coccoides* GA1 strain was washed with PBS, fixed in 2.5% glutaraldehyde buffer in 50 mM phosphate (pH 7.2), and then postfixed in 1% osmium tetroxide in 50 mM phosphate buffer (pH 7.2). Samples were dried with a critical point dryer (CPD 030; Leica Microsystems), coated with platinum, and observed under a scanning electron microscope (SU‐1510; Hitachi High‐Technologies) at 3.0 kV.

### Energy Expenditure and Fecal TG Measurements

Energy Expenditure (EE) quantification was evaluated by indirect calorimetry methods, which is estimated by the amount of O_2_ consumption and CO_2_ production during oxidation of nutrients. Briefly, male mice were maintained in a Comprehensive Lab Animal Monitoring System (Columbus Instruments, Columbus, OH, USA) according to the instructions of the manufacturer. After mice were adapted to the metabolic chamber for 6 h, volume of O_2_ consumption and CO_2_ production were continuously recorded over 24‐h period. EE quantification was calculated by an Oxymax software v5.64 (Columbus, OH, USA).

To determine the amount of fecal energy loss, the feces were collected for continuous 4 days in a single cage and monitored their food intake at the same time. After vacuum freeze‐drying, the feces were ground into powder, weighed and subjected to lipid extraction. Take 0.1 g fecal powder and add 1.2 mL methanol: chloroform (v/v, 1:2) to homogenize the power for 3 min, and then centrifuge at 3000 rpm at room temperature for 10 min. Subsequently, take 800 µL of supernatant, add 150 µL saline to mix, and then centrifuge at 3000 rpm at room temperature for 10 min. Next, take 400 µL of supernatant for air dry in the fume hood; the amount of extracted fecal TG was determined after all solvents were evaporated. Finally, fecal energy loss was rough calculated by total TG × 9 kCal g^−1^.

### 16S rDNA Gene Sequencing and Analysis

Cecal feces samples were collected, immediately frozen and stored. Genomic DNA was extracted using the E.Z.N.A. Soil DNA Kit (Omega Bio Tek; Norcross, GA, USA). The 16S rRNA gene was analyzed to evaluate the bacterial diversity using Illumina platform. Bacterial DNA was PCR amplified with barcoded universal bacterial primers targeting variable region V3+V4 of 16S rRNA gene (341F: 5′‐CCTAYGGGRBGCASCAG‐3′; 806R: 5′‐ GGACTACNNGGGTATCTAAT‐3′). Samples were sequenced with the Illumina MiSeq (PE350) or Ilumina HiSeq2500 platform (PE250). Raw fastq files were demultiplexed and quality‐filtered using QIIME2 (version 2020.2). Then the high‐quality sequences were de‐noised using DADA2 (version 2020.2) pipeline with recommended parameters, which obtains single nucleotide resolution based on error profiles within samples. DADA2 denoised sequences are usually called amplicon sequence variants (ASVs). To minimize the effects of sequencing depth on alpha and beta diversity measure, the number of sequences from each sample was rarefied to 4000, which still yielded an average Good's coverage of 97.90%. Taxonomic assignment of ASVs was performed using the Blast consensus taxonomy classifier implemented in QIIME2 and the SILVA 16S rRNA database (v138). Analysis of the 16S rRNA microbiome sequencing data was performed using the free online platform of Majorbio Cloud Platform (www.majorbio.com).

### Metagenomic Shotgun Sequencing and Analysis

The extracted microbial DNA was processed to construct metagenome shotgun sequencing libraries with insert sizes of 400 bp by using Illumina TruSeq Nano DNA LT Library Preparation Kit. Each library was sequenced by Illumina HiSeq X‐ten platform (Illumina, USA) with PE150 strategy at Personal Biotechnology Co., Ltd. (Shanghai, China). Raw sequencing reads were processed to obtain quality‐filtered reads for further analysis. Taxonomical classifications of metagenomics sequencing reads from each sample were performed using Kraken2 against a RefSeq‐derived database. Megahit (v1.1.2) was used to assemble for each sample using the meta‐large preset parameters. The generated contigs (longer than 200 bp) were then pooled together and clustered using mmseqs2 with “easy‐linclust” mode, setting sequence identity threshold to 0.95 and covered residues of the shorter contig to 90%. The lowest common ancestor taxonomy of the non‐redundant contigs was obtained by aligning them against the NCBI‐nt database by mmseqs2 with “taxonomy” mode, and contigs assigned to Viridiplantae or Metazoa were dropped in the following analysis. MetaGeneMark was used to predict the genes in the contigs. CDS sequences of all samples were clustered by mmseqs2 with “easy‐cluster” mode, setting protein sequence identity threshold to 0.90 and covered residues of the shorter contig to 90%. To assess the abundances of these genes, the high‐quality reads from each sample were mapped onto the predicted gene sequences using salmon in the quasi‐mapping‐based mode with “–meta–minScoreFraction = 0.55”, and the CPM (copy per kilobase per million mapped reads) was used to normalize abundance values in metagenomes. The functionality of the non‐redundant genes was obtained by annotated using mmseqs2 with the “search” mode against the protein databases of KEGG, EggNOG and CAZy databases, respectively. Based on the taxonomic and functional profiles of non‐redundant genes, LEfSe (Linear discriminant analysis effect size) was performed to detect differentially abundant taxa and functions across groups using the default parameters.

### Bacterial Genome *de novo* SEQUENCING

Genomic DNA of *Blautia coccoides* GA1 strain was extracted using Wizard Genomic DNA Purification Kit (Promega) according to manufacturers’ protocol. Purified genomic DNA was quantified by TBS‐380 fluorometer (Turner BioSystems Inc., Sunnyvale, CA). High quality DNA (OD260/280 ≈1.8–2.0, >1 µg) was used to do further research. For Illumina sequencing, at least 1 µg genomic DNA was used for each strain in sequencing library construction. DNA samples were sheared into 400–500 bp fragments using a Covaris M220 Focused Acoustic Shearer following manufacture's protocol. Illumina sequencing libraries were prepared from the sheared fragments using the NEXTflex Rapid DNA‐Seq Kit. Briefly speaking, 5′ prime ends were first end‐repaired and phosphorylated. Next, the 3′ ends were A‐tailed and ligated to sequencing adapters. The third step is to enrich the adapters‐ligated products using PCR. The prepared libraries then were used for Illumina sequencing PE150 bp on an Illumina HiSeq X Ten machine. The original image data is transferred into sequence data via base calling, which is defined as raw data or raw reads and saved as FASTQ file. Those FASTQ files are the original data provided for users, and they include the detailed read sequences and the read quality information. A statistic of quality information was applied for quality trimming, by which the low‐quality data can be removed to form clean data. Assemblies of the clean reads were performed using SOAPdenovo2. Glimmer was used for CDS prediction, tRNA‐scan‐SE was used for tRNA prediction and Barrnap was used for rRNA prediction. The predicted CDSs were annotated from NR, Swiss‐Prot, Pfam, GO, COG, and KEGG database using sequence alignment tools such as BLAST, Diamond and HMMER. Briefly, each set of query proteins were aligned with the databases, and annotations of best‐matched subjects (e‐value < 10^−5^) were obtained for gene annotation. Phylogenetic trees were constructed based on the Mash distance using neighbor‐joining method.^[^ ^65^ ^]^ All analyses were performed using I‐Sanger Cloud Platform (www.i‐sanger.com) from Shanghai Majorbio.

### Untargeted Metabolomics Profiling

Serum sample (100 µL) and prechilled methanol (400 µL) were mixed by well vortexing, and then were centrifuged at 15 000 g, 4 °C for 10 min. The supernatant was injected into the LC‐MS/MS system analysis (Novogene Bioinformatics Technology Co., Ltd.). Data dependent acquisition parameters are as follows: collision energies (20, 40, and 60 eV); topN precursors (10); MS1 resolution (60 000); MS1 automatic gain control (3 × 10^6^); MS2 resolution (15 000); MS2 automatic gain control (2 × 10^5^); isolation width (1.5 *m/z*); apex trigger (2 to 12 s); dynamic exclusion (10.0 s); microscans (1); isotopic exclusion (on); monoisotopic selection (off). LC‐MS/MS analyses were performed using a Vanquish UHPLC system (Thermo Fisher) coupled with an Orbitrap Q Exactive series mass spectrometer (Thermo Fisher). Samples were injected onto a Hyperil Gold column (100 × 2.1 mm, 1.9 µm) using a 16 min linear gradient at a flow rate of 0.2 mL min^−1^. The eluents for the positive polarity mode were eluent A (0.1% FA in Water) and eluent B (Methanol).The eluents for the negative polarity mode were eluent A (5 mM ammonium acetate, pH 9.0) and eluent B (Methanol).The solvent gradient was set as follows: 2% B, 1.5 min; 2–100% B, 12.0 min; 100% B, 14.0 min;100‐2% B, 14.1 min;2% B, 17 min. Q Exactive series mass spectrometer was operated in positive/negative polarity mode with spray voltage of 3.2 kV, capillary temperature of 320 °C, sheath gas flow rate of 35 arb and aux gas flow rate of 10 arb.

The raw data files generated by UHPLC‐MS/MS were processed using the Compound Discoverer 3.1 (CD3.1, Thermo Fisher) to perform peak alignment, peak picking, and quantitation for each metabolite. The main parameters were set as follows: retention time tolerance, 0.2 min; actual mass tolerance, 5 ppm; signal intensity tolerance, 30%; signal/noise ratio, 3; and minimum intensity, 100 000. After that, peak intensities were normalized to the total spectral intensity. The normalized data was used to predict the molecular formula based on additive ions, molecular ion peaks and fragment ions. And then peaks were matched with the mzCloud, mzVault, and MassList database to obtain the accurate qualitative and relative quantitative results. Notably, mzCloud database, which contains high‐resolution, accurate‐mass MS and MS/MS data, is created using commercially available standards that are individually analyzed using Thermo Orbitrap mass spectrometer. mzVault is a local database, which contains MS/MS data provided by Thermo or collected from other public databases that match the Orbitrap mass spectrometer. Whereas, MassList is a local database just containing MS data. Statistical analyses were performed using the statistical software R (R version R‐3.4.3), Python (Python 2.7.6 version) and CentOS (CentOS release 6.6), When data were not normally distributed, normal transformations were attempted using of area normalization method. Partial least squares discriminant analysis (PLS‐DA) was performed at metaX (a flexible and comprehensive software for processing metabolomics data). We applied univariate analysis (t‐test) to calculate the statistical significance (*p*‐value). The metabolites with VIP > 1 and *p*‐value ≤ 0.05 were differential metabolites. Volcano plots were used to filter metabolites of interest which based on log_2_(FC) and ‐log10(*P*‐value) of metabolites.

### Tryptophan Metabolite Incubation

Metabolite analysis in culture supernatant was performed based on an established protocol.^[^ ^19^ ^]^ 20 mL logarithmic *B. coccoides* was centrifuged at 4000 rpm for 10 min and washed twice with 20 mL pre‐reduced isotonic potassium phosphate (IPP) buffer (40 mM potassium phosphate, 10 mM MgSO_4_, pH 7.0). The final pellets were resuspended in 4 mL (1/5 culture volume) IPP buffer and incubated at room temperature for 45 min. Next, add tryptophan (at the final concentration of 10 µM) and incubate the cell suspensions at 37 °C. The cell suspensions at 0, 0.5, 1, 2, 4, and 8 h were collected and centrifuged. The supernatants were used for subsequent metabolite analyses.

### Mass Spectrometry Analysis

Serum samples (100 µL) and prechilled methanol (400 µL) were mixed by well vortexed. Cecal contents, liver, and adipose tissues (≈10 mg) were individually grounded with liquid nitrogen and the homogenate was resuspended with prechilled 80% methanol and 0.1% formic acid by well vortexed. The samples were incubated on ice for 5 min and then were centrifuged at 15 000 rpm, 4 °C for 5 min. The supernatants were diluted to final concentration containing 53% methanol by LC‐MS/MS grade water. The samples were subsequently transferred to a fresh Eppendorf tube and then were centrifuged at 15 000 g, 4 °C for 10 min. The supernatant was analyzed for tryptophan, I3AA, and other indole derivatives using 4000 Q TRAP LC‐MS/MS (AB Sciex) analysis according to a previously described method (Table S5, Supporting Information).^[^ ^33^ ^]^ Specifically, L‐tryptophan (T0254, Sigma), I3AA (45533, Sigma), indole (442619, Sigma), 3‐indoleacrylic acid (I2273, Sigma), indole‐3‐carboxaldehyde (129445, Sigma), and indole‐3‐lactic acid (I157602, Aladdin) were used as standards in the current study. The absolute concentration of metabolites was calculated according to the standard curves, which were created using six appropriate serial dilutions of the corresponding standards.

### Quantitative Real‐Time PCR

Total RNA was prepared from frozen tissues or cells with TRIZOL reagent (Invitrogen). cDNA was synthesized from 2 µg total RNA with a Reverse Transcription Kit. qRT‐PCR was carried out using SYBR Green Master Mix (Invitrogen, USA) by an QuantStudio 6 system (Applied Biosystems, Foster City, CA, USA). Cycle numbers of both GAPDH (as an internal control) and interested genes at a specific threshold within the exponential amplification range were used to calculate the relative expression of interested genes. The qRT‐PCR primers are summarized in Table S6, Supporting Information.

### Absolute Quantification of the *B. coccoides* Abundance

≈0.1 g of fecal samples was placed in a 2‐mL collection tube. Total DNA was extracted by DNA extraction kit (Qiagen, Germany). Bacterial DNA was determined by RT‐PCR as described previously.^[^ ^66^ ^]^ 16S rDNA PCR reactions were performed with the following primers: *B. coccoides* (forward: 5′‐CGGTACCTGACTAAGAAGC‐3′; reverse: 5′‐AGTTTCATTCTTGCGAACG‐3′);^[^ ^67^ ^]^ total 16S rDNA (Universal bacteria) (forward: 5′‐TCCTACGGGAGGCAGCAGT‐3′; reward: 5′‐GGACTACCAGGGTATCTAATCCTGTT‐3′) were used as an endogenous control to normalize loading between samples. Standard curves were created using fivefold serial dilution of pure culture *B. coccoides* genomic DNA. For RT‐PCR with *B. coccoides*, the standard curve employed genomic DNA in the following amounts per reaction (in pg): 200, 40, 8, 1.6, 0.32, and 0.064 (R^2^ = 0.9967). For qPCR with total 16S rDNA, the standard curve employed genomic DNA in the following amounts per reaction (in ng): 5, 1, 0.2, 0.04, 0.008, and 0.0016 (R^2^ = 0.9993). All reactions were performed in duplicate, with the mean value used for statistical analyses. According to the standard curve, the amount of total DNA, and the fecal weight, we can calculate the amount of *B. coccoides* genomic DNA/g fecal. Next, use the following formulas to convert 1 ng *B. coccoides* genomic DNA to comparable genome equivalents: genome equivalents/ng DNA = Avogadro's number/(genome size × 1e9 × average molecular weight of nucleotides). The Avogadro's number is 6.0221 × 10^23^ copy number/mol; for *B. coccoides*, the genome size is 6.22 Mbp; the average molecular weight of nucleotide is 660 g mol^−1^ bp^−1^. In the end, *B. coccoides* genome equivalents/g fecal = (the amount of genomic DNA/g fecal) × (genome equivalents/ng DNA). Therefore, *B. coccoides* was assigned a multiplier of 1.46 × 10^5^ genome equivalents/ng DNA based on its genome size (6.22 Mbp; DSM935); the gut microbial community as a whole was assigned a multiplier of 2.03 × 10^5^/ng DNA based on an average genome size of 4.50 Mbp.

### Western Blot

Proteins from tissues and cells were prepared using RIPA buffer. Protein samples were subjected to concentration determination and immunoblot assay with the following antibodies: phosphorylated (p)‐IR (Tyr 1150/1151; 3024S), total IR (3025), p‐AKT (Ser 473; 9271S), total AKT (9272S), p‐GSK3β (Ser 9; 9336S), and total GSK3β(9315S) from Cell Signaling Technology; AhR (17840‐1‐AP) and β–Actin (81115‐1‐RR) from Proteintech; FGF21 (ab171941) and UCP1 (ab209483) from Abcam. The specific proteins were visualized by ECL Plus (Amersham Biosciences). Band intensities were measured using Quantity One (Bio‐Rad Laboratories) and normalized to actin or their correspondent total protein.

### Statistical Analysis

Statistical analysis was performed using GraphPad Prism version 8.0 (San Diego, CA, USA). All values were presented as means ± SEM. Differences between two treatment groups were assessed using two‐tailed unpaired Student's *t*‐test; For comparisons between more than two groups, one‐way or two‐way analysis of variance (ANOVA) followed by Tukey's *post hoc* test or Dunnett's *post hoc* test was used. Spearman's correlation analysis was performed to analyze the correlation of abundance of specific genus and metabolic parameters. For GTT and ITT, the difference between multiple curves is indicated by the AUC. Significant differences emerging from the above tests are indicated in the Figures by *p < 0.05, **p < 0.01, and ***p < 0.001. Notable near‐significant differences (0.05 < *p* < 0.1) are indicated in the Figures. Notable non‐significant (and non‐near significant) differences are indicated in the Figures by “‘n.s.”’.

## Conflict of Interest

The authors declare no conflict of interest.

## Author contributions

Y.N., X.H., and Y.S. contributed equally to this work. F.G., Y.N., and X.H. planned and supervised the experimental work and data analysis; Y.N., X.H., P.L., F.J., S.N., and H.J. performed all the experiments; Y.S., J.X., and C.W. recruited human subjects and performed the related data analysis; X.H., Y.N, J.Q., S.C., Q.Z., and F.G. analyzed the data; X.H., W.J., S.Y., J.C., and R.H. cultured the anaerobes; and X.H., Y.N. and F.G. wrote the manuscript; F.G. conceived and supervised the study.

## Supporting information

Supporting Information

## Data Availability

The data that support the findings of this study are available from the corresponding author upon reasonable request.
