# Supplementary material for: Blautia Coccoides is a Newly Identified Bacterium Increased by Leucine Deprivation and has a Novel Function in Improving Metabolic Disorders
Source: Adv Sci (Weinh). 2024 Mar 1;11(18):2309255. doi: 10.1002/advs.202309255 (PMC11095201; doi:10.1002/advs.202309255)
Supplement: Supplementary file 1 — Supporting Information [file ADVS-11-2309255-s001.pdf]

ADVANCED SCIENCE
Open Access

Supporting Information

for *Adv. Sci.*, DOI 10.1002/advs.202309255

Blautia Coccoides is a Newly Identified Bacterium Increased by Leucine Deprivation and has a Novel Function in Improving Metabolic Disorders

Yuguo Niu, Xiaoming Hu, Yali Song, Cunchuan Wang, Peixiang Luo, Shihong Ni, Fuxin Jiao, Ju Qiu, Weihong Jiang, Sheng Yang, Jun Chen, Rui Huang, Haizhou Jiang, Shanghai Chen, Qiwei Zhai, Jia Xiao and Feifan Guo**

Supporting Information

Blautia coccoides is a newly identified bacterium increased by leucine deprivation and has a novel function in improving metabolic disorders

Yuguo Niu [1,*], Xiaoming Hu [1,*], Yali Song [2,*], Cunchuan Wang [2], Peixiang Luo [3], Shihong Ni [1], Fuxin Jiao [3], Ju Qiu [3], Weihong Jiang [4], Sheng Yang [4], Jun Chen [4], Rui Huang [3], Haizhou Jiang [1], Shanghai Chen [1], Qiwei Zhai [3], Jia Xiao [2,#], Feifan Guo [1,#]

*These authors contributed equally

# To whom Correspondence should be addressed.

Email: ffguo@fudan.edu.cn (Feifan Guo); edwinsiu@connect.hku.hk (Jia Xiao)

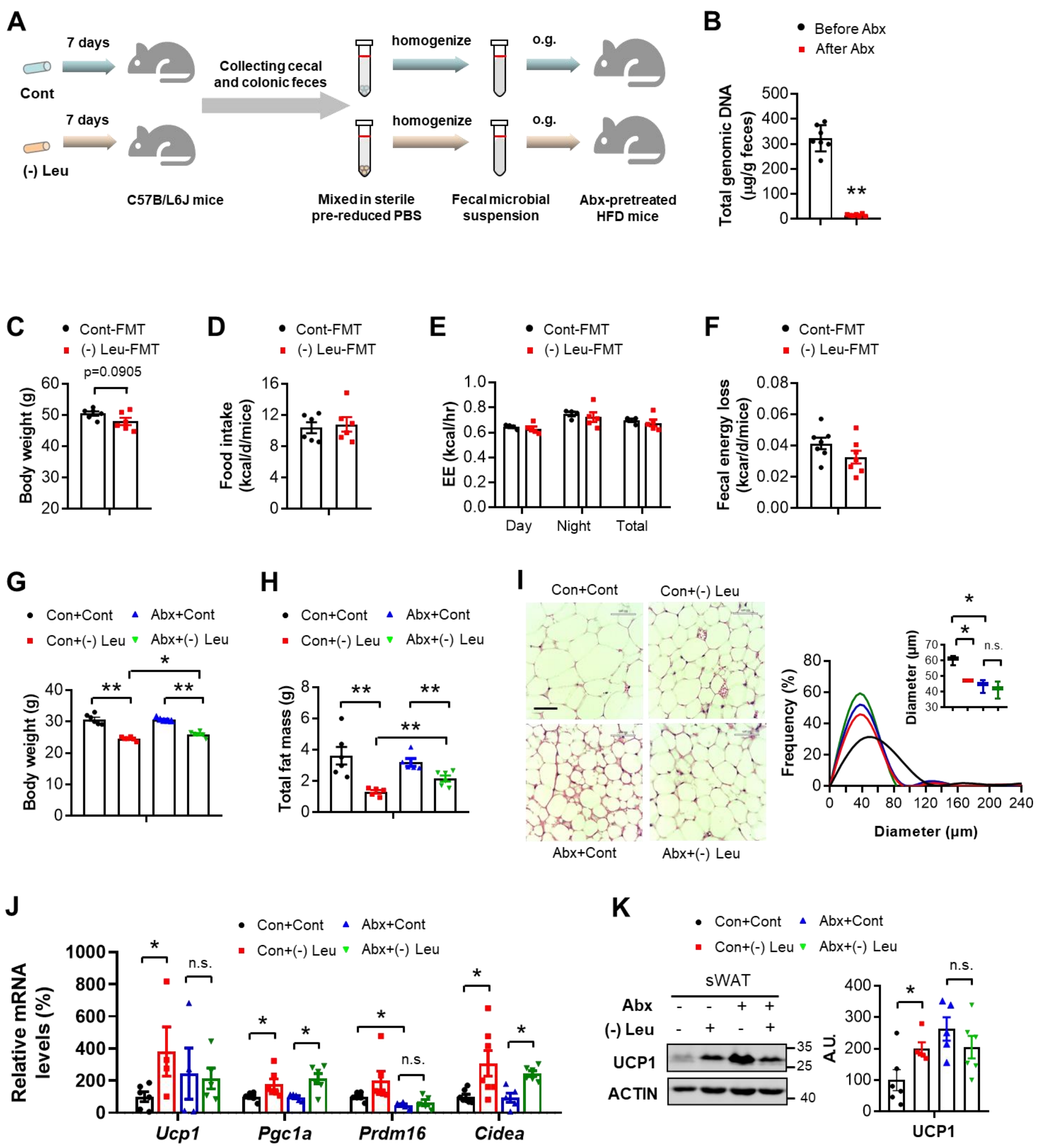

Figure S1. The effects of FMT in metabolism and the resistance of leucine deprivation-mediated metabolic improvements by antibiotics.

(A–F) HFD mice were given with antibiotic cocktail (termed as Abx) in drinking water. After 4 weeks, the Abx mice were transplanted with the fecal microbiota from control diet (Cont-FMT) or leucine-deprived diet ((-) Leu-FMT) donors for 30 days (n = 5–7 biological replicates per group). o.g.: oral gavage. (A) The FMT procedures. (B) The DNA contents of cecal microbiota before or after Abx treatment. (C) Body weight. (D) Food intake. (E) Energy expenditure (EE). (F) Fecal energy loss. (G–K) 10-week-old male C57BL/6J WT mice were given Abx in drinking water or given autoclaved water (Con). After 4 weeks, the Con

or Abx mice were treated with the (-) Leu or Cont diet for 7 days, respectively (n=5–8 biological replicates per group). (G) Body weight. (H) Total fat mass. (I) The H&E staining of sWAT. Scale bars, 50 μm. The right panel is the frequency distribution of adipocyte cell size in sWAT and the box plot is average adipocyte diameter. (J) Real-time PCR analysis of browning related genes (*Ucp1*, *Pgc1α*, *Prdm16*, and *Cidea*) in sWAT. (K) Western blot analysis of UCP1 protein levels. The right panel is the densitometry analysis of UCP1 protein levels. A.U.: arbitrary units. All values are expressed as the mean ± SEM. Statistical comparisons were carried out by unpaired two-tailed Student's *t* test or two-way ANOVA; *$p < 0.05$, **$p < 0.01$, and n.s.: no significance.

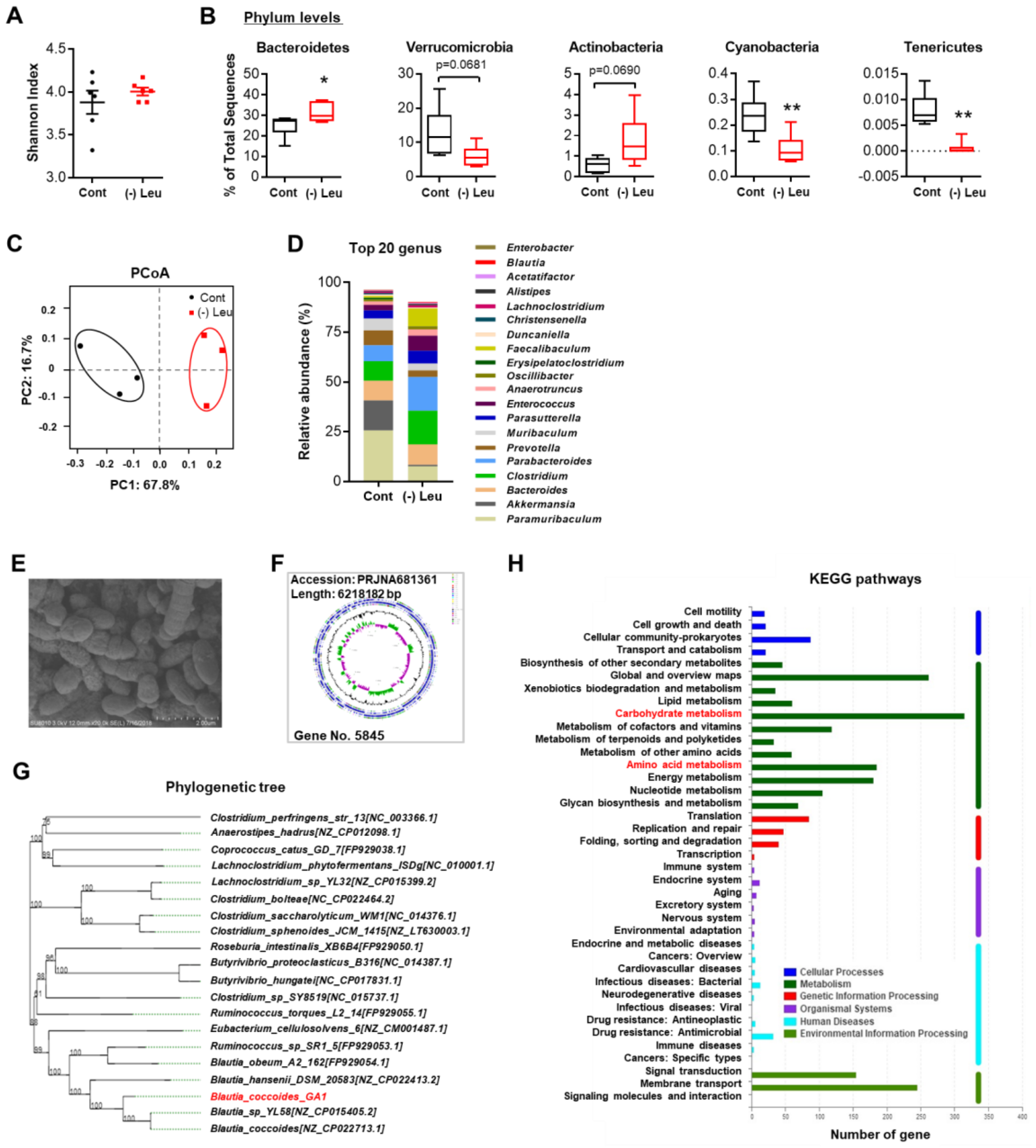

Figure S2. 16S rDNA and metagenomic sequencing.

(A–D) 10-week-old male C57BL/6J WT mice were treated with leucine deprivation diet ((-) Leu) or control diet (Cont) for 7 days. The cecal microbiota were collected for bacterial 16S rDNA (A and B, n=6 biological replicates per group) and metagenomic sequencing (C and D, n=3 biological replicates per group). (A) Shannon index analysis of effective reads. (B) The relative abundance of Bacteroidetes, Verrucomicrobia, Actinobacteria, Cyanobacteria, and Tenericutes at the phylum level. (C) Principal-coordinate analysis (PCoA) of the gut microbiota structure based on Bray-Curtis distance. (D) Bacterial taxonomic profiling in the top 20 bacteria at genus level. (E) Scanning electron

microscopy of *B. coccoides* GA1. (F–H) Whole-genome sequence of *B. coccoides* GA1 was determined by *de novo* sequencing. (F) Complete genome graph of *B. coccoides* GA1. From inner to outer: GC skew, GC content, tRNA/rRNA, CDS (reverse and forward strand). (G) Phylogenetic tree analysis of *B. coccoides* GA1. (H) The Kyoto Encyclopedia of Genes and Genomes (KEGG) pathway analysis of the encoded genes of *B. coccoides* GA1. All values are expressed as the mean ± SEM. Statistical comparisons were carried out by nonparametric Mann-Whitney *U* test; *$p < 0.05$ and **$p < 0.01$.

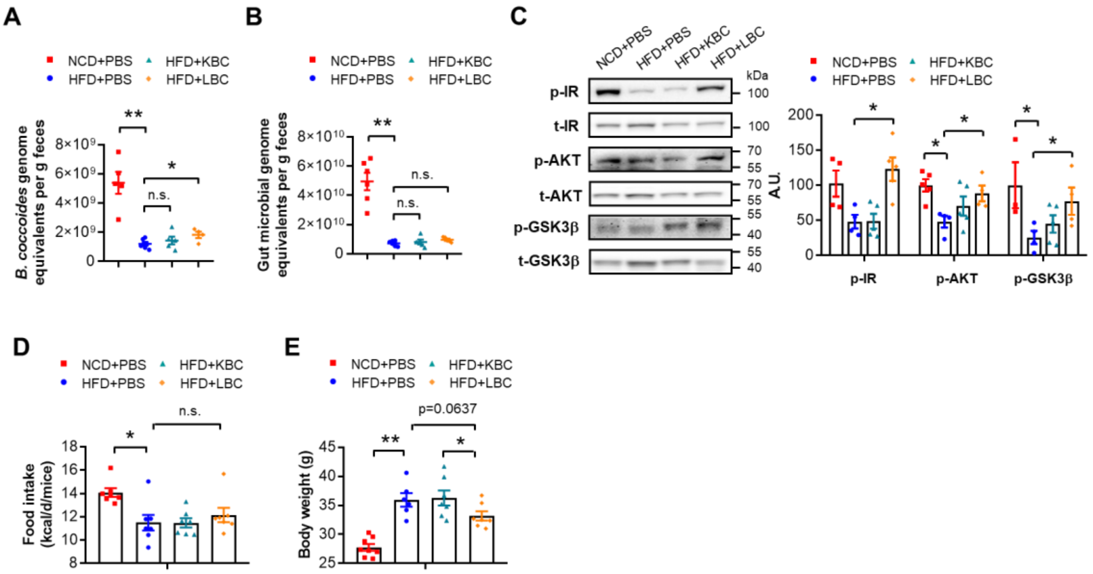

Figure S3. The metabolic effect of *B. coccoides*.

NCD and HFD-fed mice were orally gavage with PBS, heat-killed *B. coccoides* GA1 (KBC), or live *B. coccoides* GA1 (LBC) for 8 weeks, respectively (n = 6–8 biological replicates per group). (A, B) Absolute abundance of *B. coccoides* or total microbial DNA per gram of feces. (C) Western blot analysis of p-IR, p-AKT, and p-GSK3β levels in liver. The right panel is the densitometry analysis of the relative abundance of phosphorylated proteins normalized to their total protein levels. A.U.: arbitrary units. (D) Food intake. (E) Body weight. All values are expressed as the mean ± SEM. Statistical comparisons were carried out by one-way ANOVA; *$p < 0.05$, **$p < 0.01$, and n.s.: no significance.

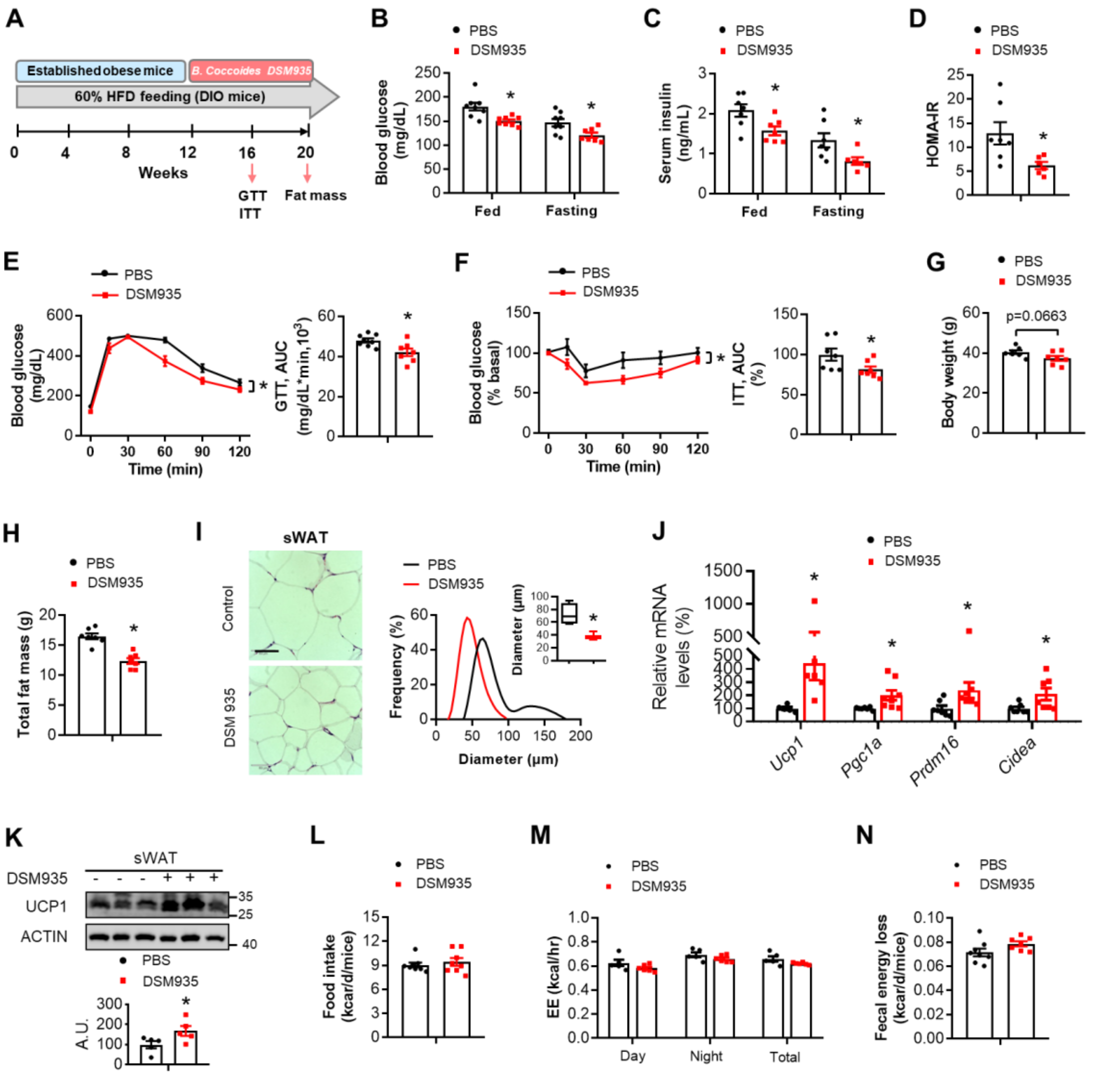

Figure S4. Oral administration of *B. coccoides* DSM935 improves metabolic disorders in HFD mice.

HFD mice were either orally gavage with PBS or live *B. coccoides* DSM935 strain (DSM935) for 8 weeks (n = 6–8 biological replicates per group). (A) Experimental procedure. (B) Fed and fasting blood glucose levels. (C) Fed and fasting serum insulin levels assayed by ELISA. (D) HOMA-IR index. (E) Glucose tolerance tests. The right panel is AUC. (F) Insulin tolerance tests (0.75 U/kg). The right panel is the AUC. (G) Body weight. (H) Total fat mass. (I) The H&E staining of sWAT. Scale bars, 50 μm. The right panel is the frequency distribution of adipocyte cell size in sWAT and the box plot is average adipocyte diameter. (J) Real-time PCR analysis of browning related genes (*Ucp1*, *Pgc1α*, *Prdm16*, and *Cidea*) in sWAT. (K) Western blot analysis

of UCP1 protein levels. The bottom panel is the densitometry analysis of UCP1 protein levels. A.U.: arbitrary units. (L) Food intake. (M) Energy expenditure (EE). (N) Fecal energy loss. All values are expressed as the mean ± SEM. Statistical comparisons were carried out by unpaired two-tailed Student's *t* test; *$p < 0.05$.

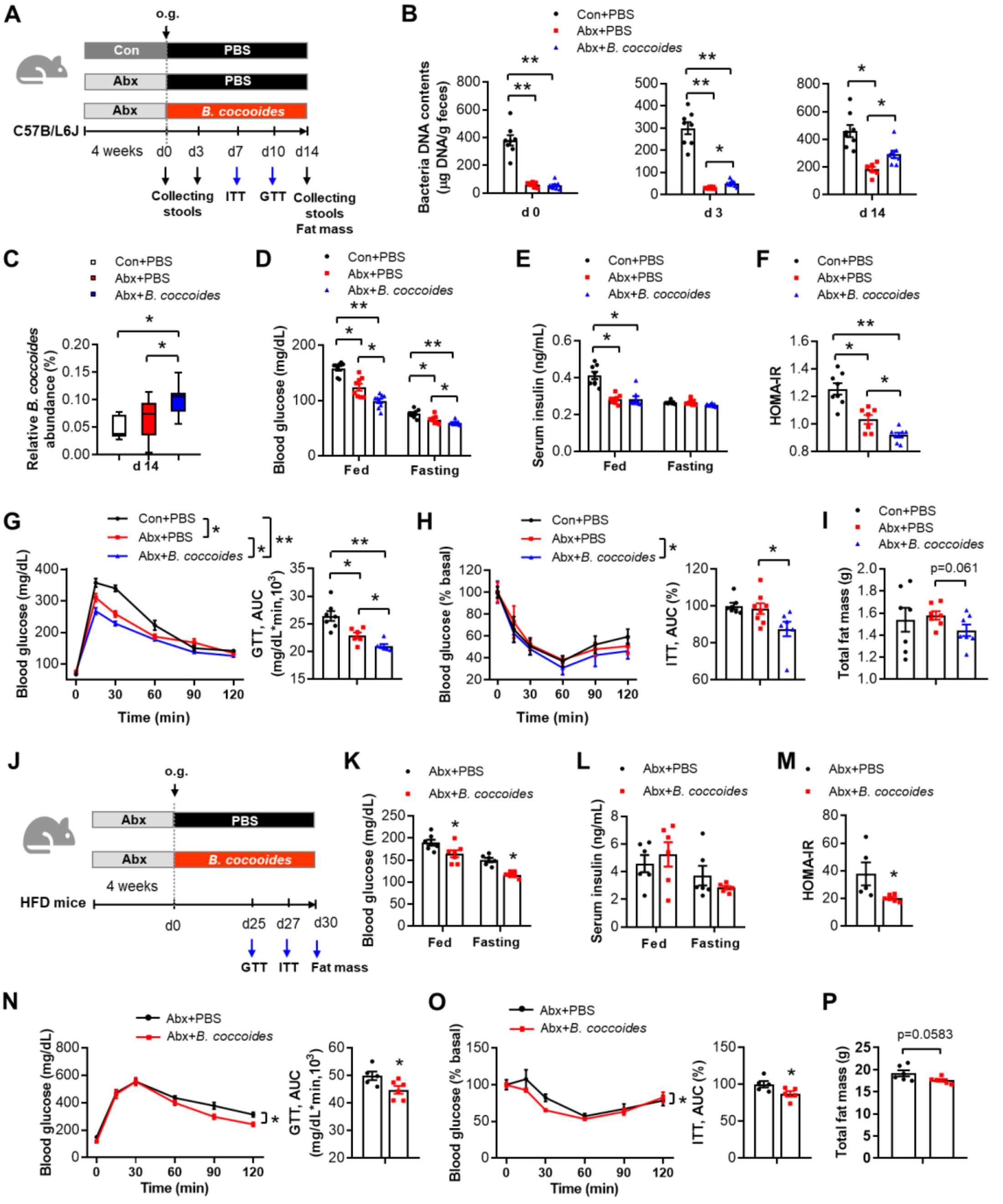

Figure S5. Colonization of *B. coccoides* in gnotobiotic mice improves insulin sensitivity.

(A–I) 10-week-old male C57BL/6J WT mice were given antibiotic cocktail (Abx) in drinking water or given autoclaved water (Con). After 4 weeks, the Con or Abx mice were gavage with PBS or *B. coccoides*, respectively (n=7–9 biological replicates per group). o.g.: oral gavage. (A) Experimental procedures. (B) The DNA contents of cecal microbiota at day 0, 3, and 14 after Abx treatment, respectively. (C) The relative abundance of *B. coccoides* at day

14. (D) Fed and fasting blood glucose levels. (E) Fed and fasting serum insulin levels assayed by ELISA. (F) HOMA-IR index. (G) Glucose tolerance tests. The right panel is the AUC. (H) Insulin tolerance tests (0.5 U/kg). The right panel is the AUC. (I) Total fat mass. (J–P) HFD mice were given with Abx in drinking water. After 4 weeks, the Abx mice were gavage with PBS or *B. coccoides*, respectively (n=5–7 biological replicates per group). (J) Experimental procedures. (K) Fed and fasting blood glucose levels. (L) Fed and fasting serum insulin levels assayed by ELISA. (M) HOMA-IR index. (N) Glucose tolerance tests. The right panel is the AUC. (O) Insulin tolerance tests (1 U/kg). The right panel is the AUC. (P) Total fat mass. All values are expressed as the mean ± SEM. Statistical comparisons were carried out by unpaired two-tailed Student's *t* test; $*p < 0.05$ and $**p < 0.01$.

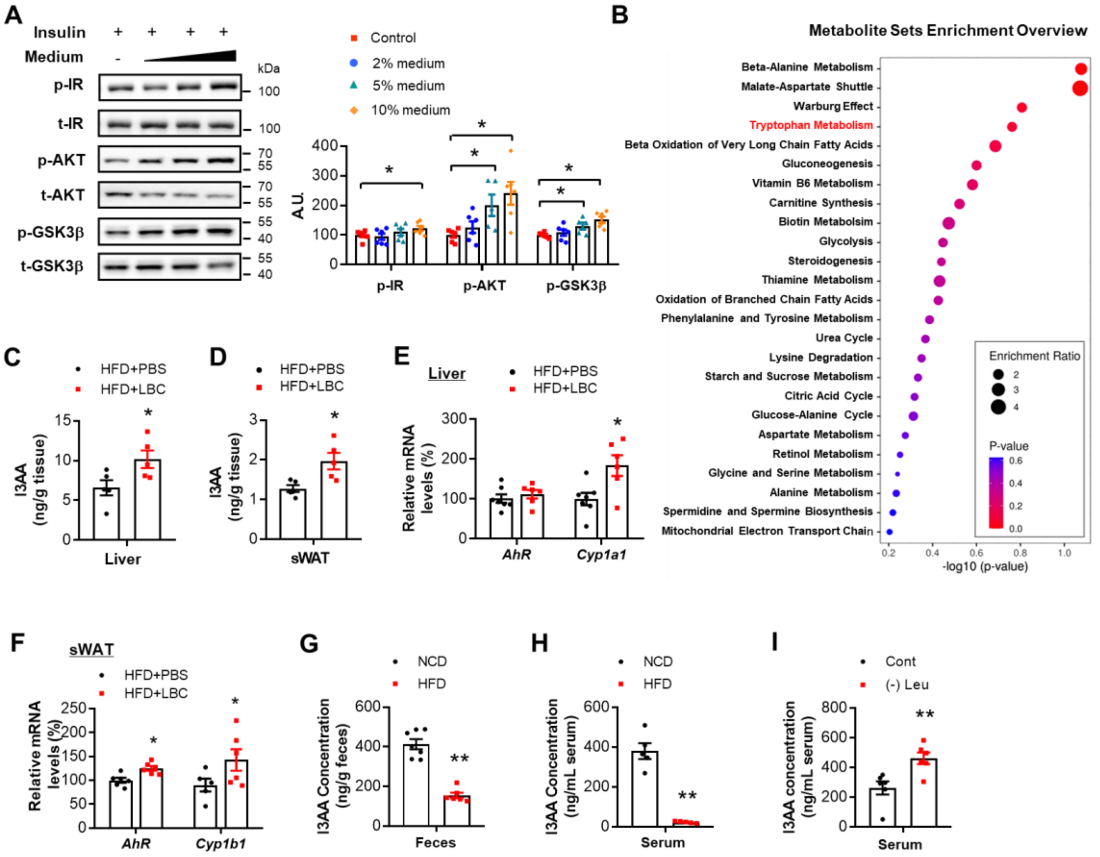

Figure S6. *B. coccoides* metabolites enrichment, I3AA levels, and AhR targeted gene expression.

(A) The growth media of *B. coccoides* after 48 h culture was collected to incubate primary hepatocytes for 48 h at indicate concentrations, and then stimulated with 100 nM insulin for 20 min (n=5–6 replicates per group). Western blot analysis of p-IR, p-AKT, and p-GSK3β levels in primary hepatocytes. The right panel is the densitometric analysis of the relative abundance of phosphorylated proteins normalized to their total protein levels. A.U.: arbitrary units. (B) Enrichment analysis of differential metabolites. (C–F) HFD mice were either orally gavage with PBS or live *B. coccoides* (LBC) daily for 8 weeks (n=5–7 mice per group). (C, D) I3AA levels in liver (C) or sWAT (D) detected by LC-MS/MS. (E, F) Real-time PCR analysis of *AhR*, *Cyp1a1*, or *Cyp1b1* mRNA levels in liver (E) or sWAT (F). (G, H) The I3AA levels in cecal feces (G) and sera (H) of mice fed with HFD or NCD for 16 weeks (n=5–7 biological replicates per group). (I) Serum I3AA levels in mice fed with control

diet (Cont) or leucine deprivation diet ((-) Leu) for 7 days (n=6 biological replicates per group). All values are expressed as the mean ± SEM. Statistical comparisons were carried out by unpaired two-tailed Student's *t* test; $*p < 0.05$, $**p < 0.01$.

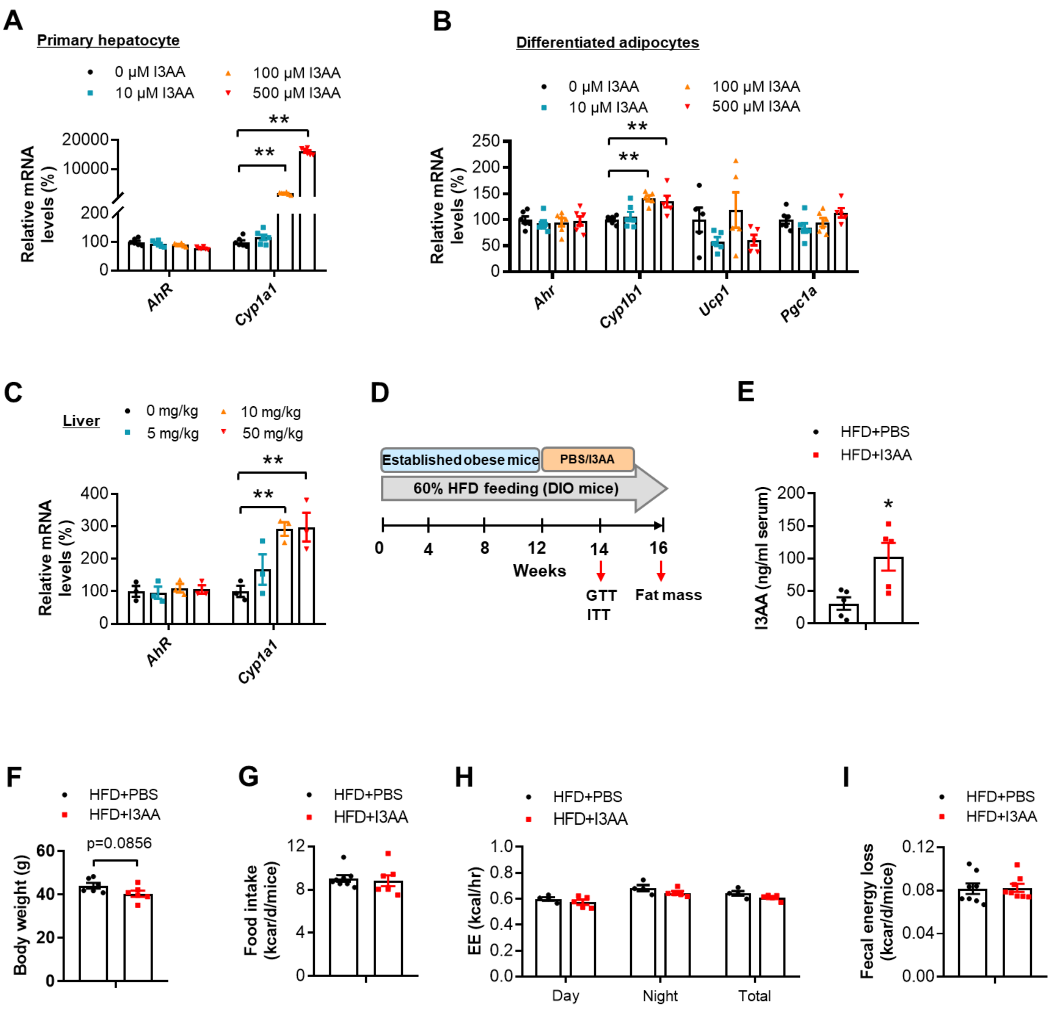

Figure S7. Metabolic effects of I3AA *in vitro* and *in vivo*.

(A, B) Primary hepatocytes (A) or differentiated adipocytes (B) were incubated with indicate dose of I3AA for 48 h. Real-time PCR analysis of *AhR* and its target gene *Cyp1a1* mRNA levels (n= 5–6 replicates per group). (C) HFD mice were oral gavage with indicated dose of I3AA for 2 weeks. Real-time PCR analysis of *AhR* and its target gene *Cyp1a1* mRNA levels in the liver (n= 3 biological replicates per group). (D–I) HFD mice were orally gavage with PBS or 10 mg/kg I3AA for 4 weeks, respectively (n=6–7 biological replicates per group). (D) Experimental procedure. (E) Serum I3AA levels detected by LC-MS/MS. (F) Body weight. (G) Food intake. (H) Energy expenditure (EE). (I) Fecal energy loss. All values are expressed as the mean ± SEM. Statistical comparisons were carried out by unpaired two-tailed Student's *t* test; *$p < 0.05$ and **$p < 0.01$.

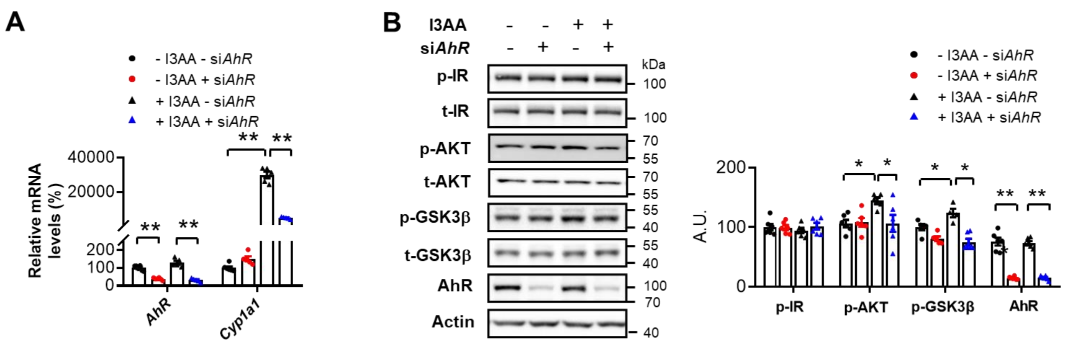

Figure S8. I3AA increased primary hepatocytes insulin signaling is depend on AhR.

Primary hepatocytes were transfected with negative control small interfering RNA (- si*AhR*) or siRNA targeting at mouse *AhR* (+ si*AhR*) for 24h, and then incubated with or without 500 μM I3AA for the next 48 hours. At last, cells were additionally incubated with 100 nM insulin for 20 min in (B) to detect insulin signaling (n= 5–6 replicates per group). (A) Real-time PCR analysis of *AhR* and *Cyp1a1* mRNA levels. (B) Western blot analysis of p-IR, p-AKT, p-GSK3β, and AhR levels. The right panel is the densitometry analysis of the relative abundance of phosphorylated proteins normalized to their total protein or Actin levels. A.U.: arbitrary units. All values are expressed as the mean ± SEM. Statistical comparisons were carried out by two-way ANOVA; *$p < 0.05$, **$p < 0.01$.

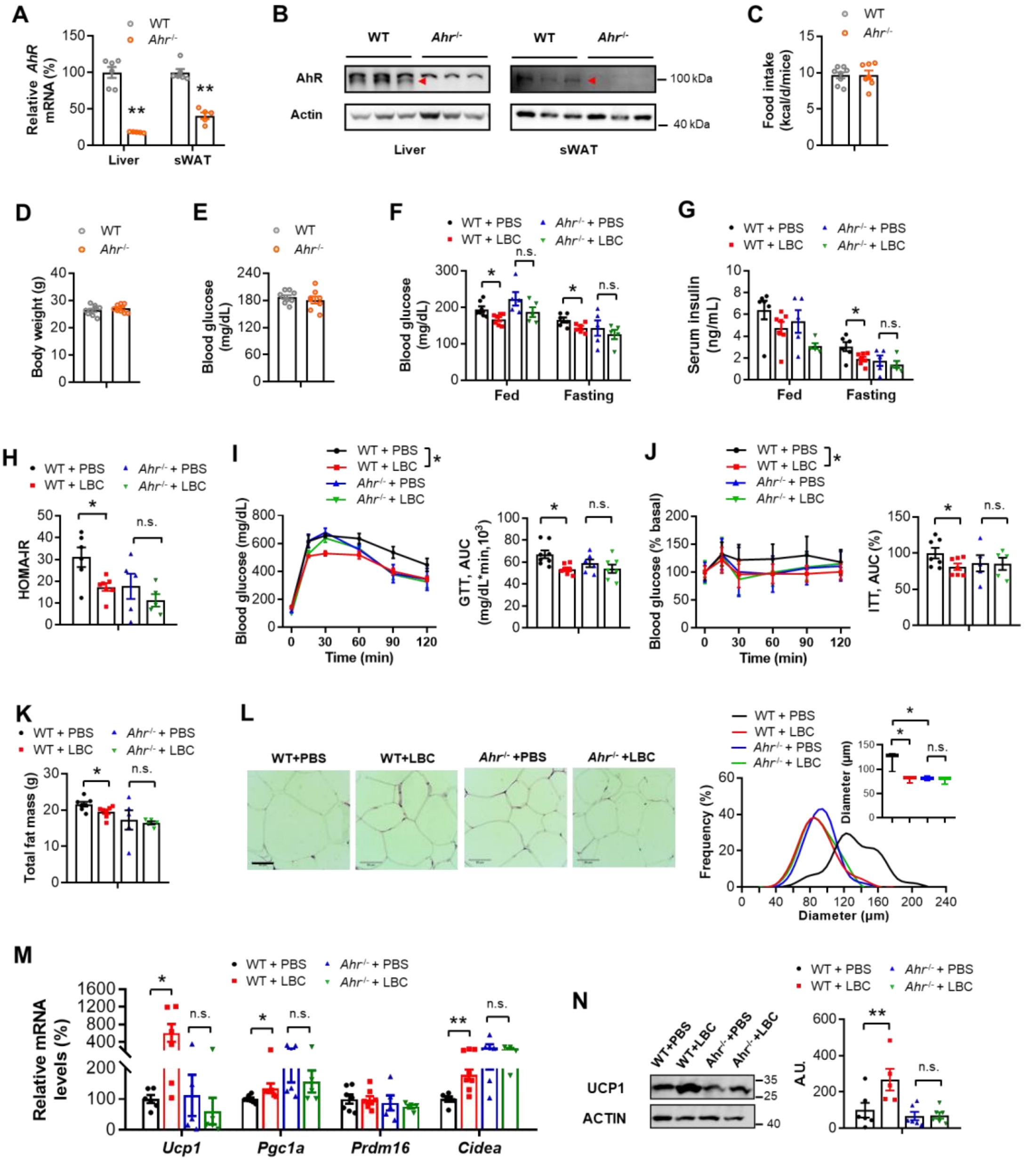

Figure S9. ***B. coccoides*** **cannot induce metabolic improvements in global** ***AhR*** **knockout mice.**

(A–E) The basic phenotypes in wild-type (WT) and *AhR* knockout (*Ahr*$^{-/-}$) mice (n=6–7 biological replicates per group). (A) Real-time PCR analysis of *AhR* mRNA levels in liver or sWAT. (B) Western blot analysis of AhR protein levels in liver or sWAT. Red arrow indicates specific protein bands of AhR. (C) Food intake. (D) Body weight. (E) Fed blood glucose levels. (F–N) WT and *Ahr*$^{-/-}$ mice were fed with HFD for 12 weeks, and then were either orally gavage with PBS or live *B. coccoides* (LBC) for 8 weeks (n=4–8 biological replicates per

group). (F) Fed and fasting blood glucose levels. (G) Fed and fasting serum insulin levels assayed by ELISA. (H) HOMA-IR index. (I) Glucose tolerance tests. The right panel is the AUC. (J) Insulin tolerance tests (1 U/kg). The right panel is the AUC. (K) Total fat mass. (L) The H&E staining of sWAT. Scale bars, 50 μm. The right panel is the frequency distribution of adipocyte cell size in sWAT and the box plot is average adipocyte diameter. (M) Real-time PCR analysis of browning related genes (*Ucp1*, *Pgc1α*, *Prdm16*, and *Cidea*) in sWAT. (N) Western blot analysis of UCP1 protein levels. The right panel is the densitometry analysis of UCP1 protein levels. A.U.: arbitrary units. All values are expressed as the mean ± SEM. Statistical comparisons were carried out by unpaired two-tailed Student's t test or two-way ANOVA; *p < 0.05, **p < 0.01, and n.s.: no significance.

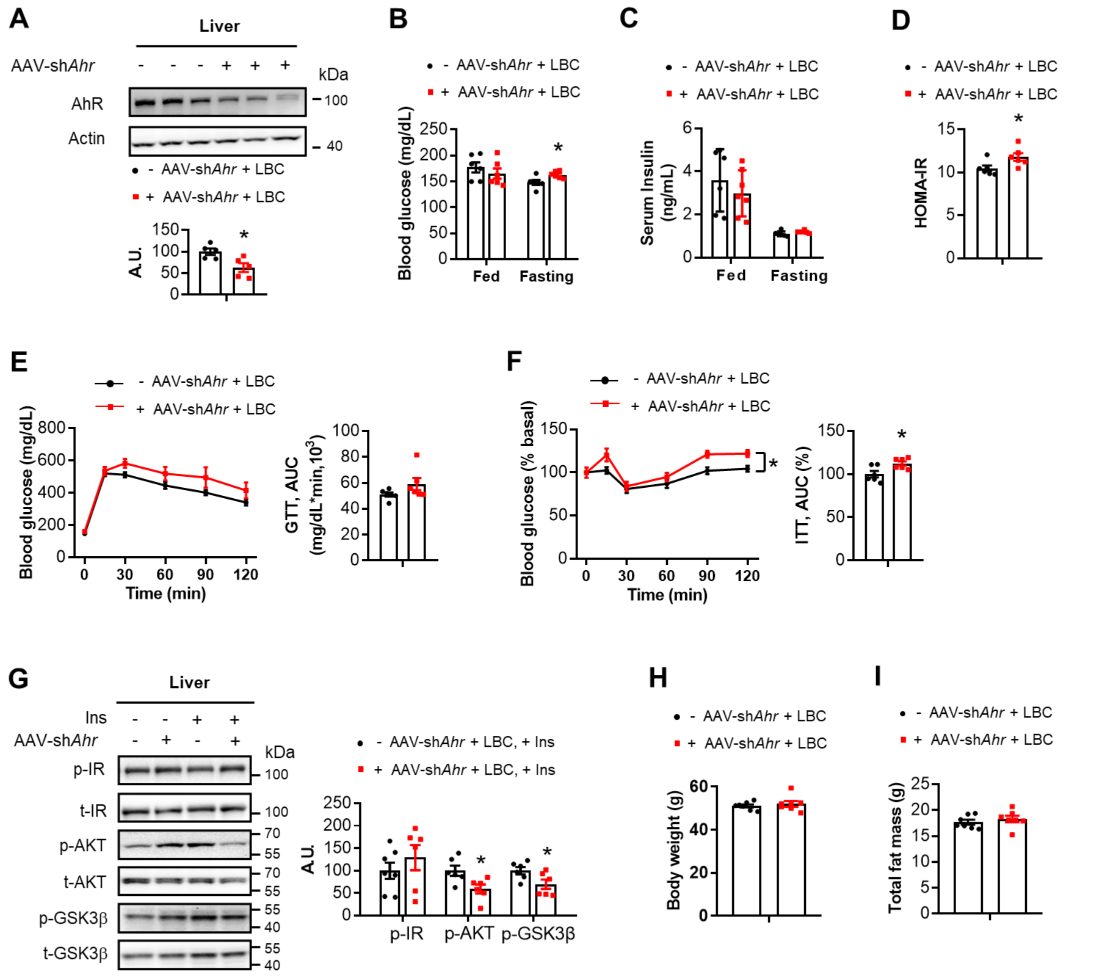

Figure S10. ***B. coccoides*** **induced improvement in insulin sensitivity is depend on liver AhR.**

HFD mice were injected with AAV-shGFP (- AAV-sh*Ahr*) or AAV-sh*Ahr* (+ AAV-sh*Ahr*) by tail vein, respectively. After 3 weeks, all mice were gavage with live *B. coccoides* (LBC) for 4 weeks (n=5–6 biological replicates per group). (A) Western blot analysis of liver AhR expression. The bottom panel is the densitometry analysis of AHR protein levels. A.U.: arbitrary units. (B) Fed and fasting blood glucose levels. (C) Fed and fasting serum insulin levels assayed by ELISA. (D) HOMA-IR index. (E) Glucose tolerance tests. The right panel is the AUC. (F) Insulin tolerance tests (0.75 U/kg). The right panel is the AUC. (G) Western blot analysis of p-IR, p-AKT, and p-GSK3β levels in liver with or without insulin stimulation. The right panel is the densitometric analysis of the relative abundance of phosphorylated proteins normalized to their total protein levels. (H) Body weight. (I) Total fat mass. All values are expressed as the

mean ± SEM. Statistical comparisons were carried out by unpaired two-tailed Student's *t* test; $*p < 0.05$.

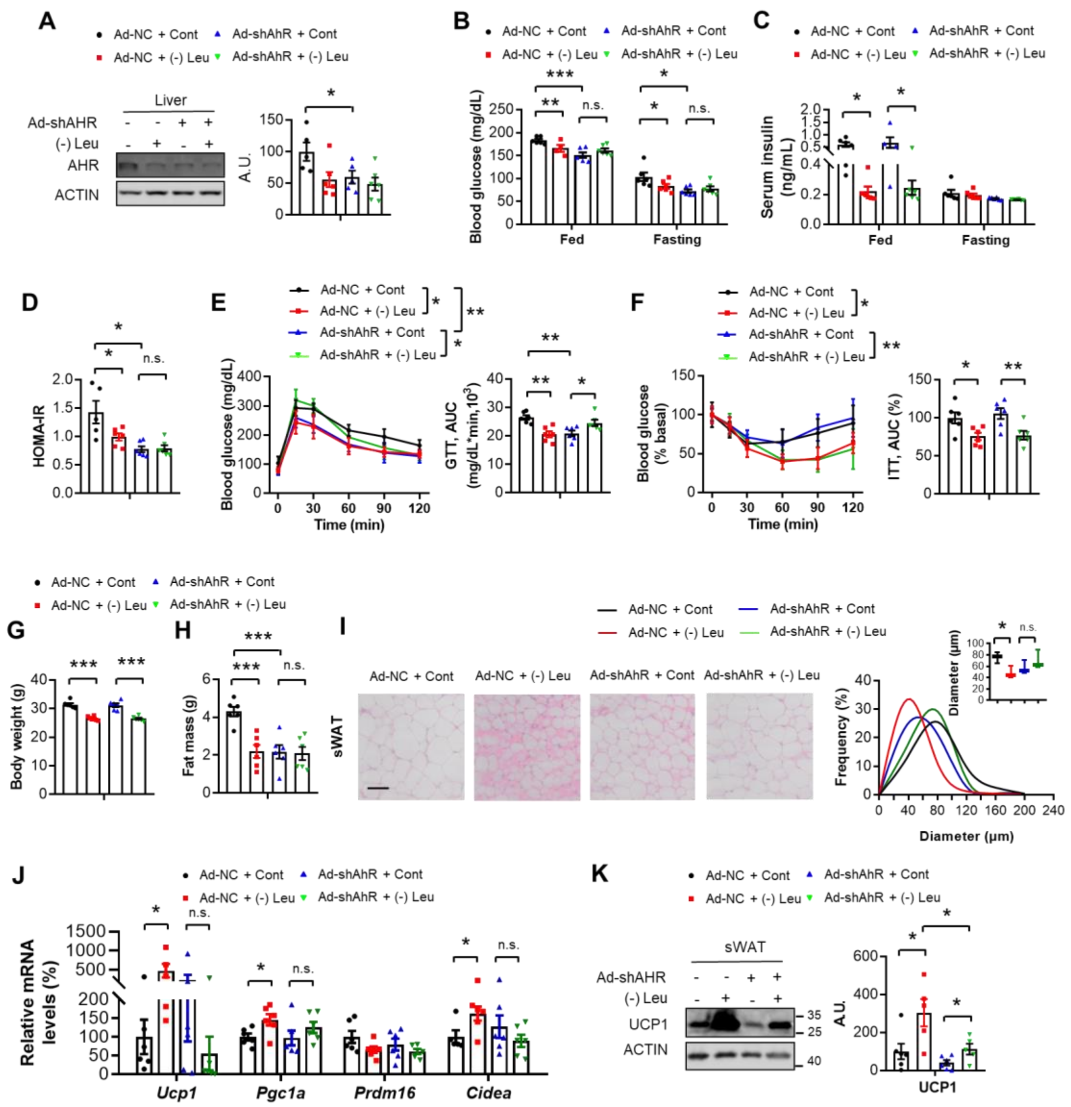

Figure S11. Leucine deprivation induced metabolic improvements are partially dependent on liver AhR.

12-week-old male C57BL/6J WT mice were injected with Ad-NC or Ad-sh*AhR* via tail vein, respectively. After 3 days, Ad-NC and Ad-sh*AhR* mice were either fed with control diet (Cont) or (-) leucine diet ((-) Leu) for another 7 days (n=5–6 biological replicates per group). (A) Western blot analysis of AhR protein levels in the liver. The right panel is the densitometry analysis of AhR protein levels. A.U.: arbitrary units. (B) Fed and fasting blood glucose levels. (C) Fed and fasting serum insulin levels assayed by ELISA. (D) HOMA-IR index. (E) Glucose tolerance tests. The right panel is AUC. (F) Insulin tolerance tests (0.5 U/kg). The right panel is the AUC. (G) Body weight. (H) Total fat mass. (I) The H&E staining of sWAT. Scale bars, 50 μm. The right panel is the

frequency distribution of adipocyte cell size in sWAT and the box plot is average adipocyte diameter. (J) Real-time PCR analysis of the gene expression related to sWAT browning, including *Ucp1*, *Pgc1α*, *Prdm16*, and *Cidea*. (K) Western blot analysis of UCP1 protein levels. The right panel is the densitometry analysis of UCP1 protein levels. All values are expressed as the mean ± SEM. Statistical comparisons were carried out by two-way ANOVA; *$p < 0.05$, **$p < 0.01$, ***$p < 0.001$, and n.s.: no significance.

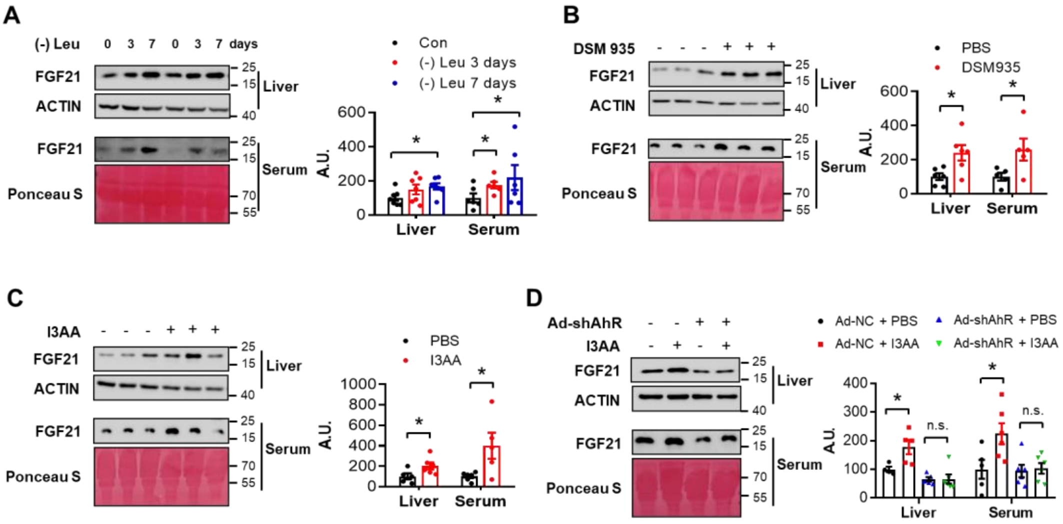

Figure S12. Leucine deprivation-*B. coccoides*-I3AA-AhR axis affects FGF21 levels both in the liver and serum.

(A) 12-week-old male C57BL/6J WT mice were fed with control (Cont) diet or leucine deprivation ((-) Leu) diet for indicated time (n=5–6 biological replicates per group). (B) HFD mice were either orally gavage with PBS or *B. coccoides* DSM935 strain for 8 weeks (n=5–6 biological replicates per group). (C) HFD mice were either orally gavage with PBS or 10 mg/kg I3AA for 4 weeks (n=5–6 biological replicates per group). (D) HFD mice injected with Ad-NC or Ad-sh*AhR* via tail vein, respectively. And then, the Ad-NC or Ad-sh*AhR* mice were either orally gavage with PBS or 10 mg/kg I3AA daily for 10 days (n=4–6 biological replicates per group). (A–D) Western blot analysis of FGF21 protein levels in the liver or serum. The right panel is the densitometry analysis of FGF21 protein levels. A.U.: arbitrary units. All values are expressed as the mean ± SEM. Statistical comparisons were carried out by unpaired two-tailed Student's t test; *p < 0.05 and n.s.: no significance.

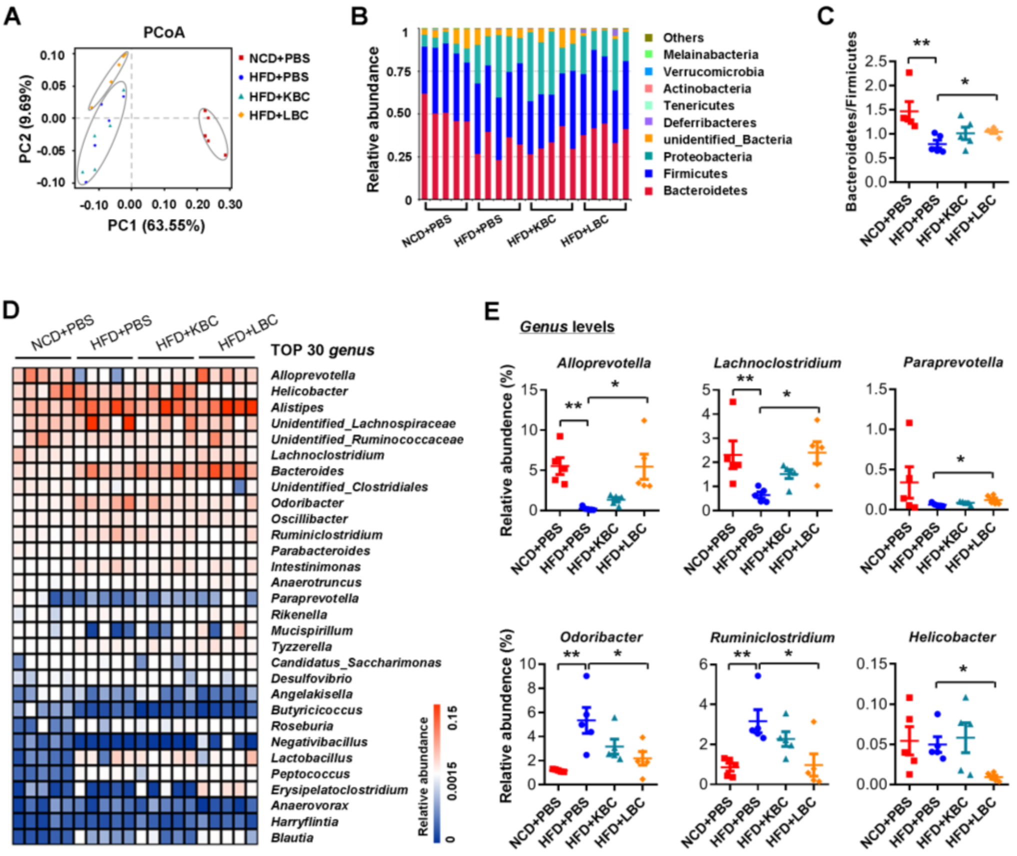

Figure S13. *B. coccoides* reshapes the structures of the gut microbiota in HFD mice.

NCD and HFD mice were either orally gavage with PBS, heat-killed B. coccoides (KBC) or live *B. coccoides* (LBC) for 8 weeks. Cecal microbiota were used for bacterial 16S rDNA sequencing (n = 5 biological replicates per group). (A) Principal-coordinate analysis (PCoA) of the microbial composition. (B) Relative abundance of obtained species. (C) Bacteroidetes:Firmicutes ratio. (D) Analysis of the top 30 bacterial genus. (E) The abundance of detailed bacteria genus. All values are expressed as the mean ± SEM. Statistical comparisons were carried out by the Kruskal–Wallis test followed by a Dunn's post hoc test; *$p < 0.05$ and **$p < 0.01$.

Supplemental Tables

Table S1. Differential metabolites

Metabolites	PBS-Mean	LBC-Mean	FC	log2FC	p-value	mzVault Best Match	mzCloud Best Match
2-[5-(2-chloro-4-fluorophenyl)-1,2,4-oxadiazol-3-yl] pyridine	61168	299808	4.90	2.29	0.0384		43.2
6β-Naloxol	375739	955477	2.54	1.35	0.0315		58.1
Methyl indole-3-acetate	21647908	51924356	2.40	1.26	0.0056	93.7	83.2
4-(3,4-dihydro-2H-1,5-benzodioxepin-7-ylamino)-4-oxobutanoic acid	615663	1287089	2.09	1.06	0.0065		42.8
Indole-3-acetic acid	1844725	3851824	2.09	1.06	0.0287	86.9	96
4-Quinolinecarboxylic acid	1276032	2507408	1.97	0.97	0.0019	58.2	
16-Hydroxyhexadecanoic acid	8326773	15134301	1.82	0.86	0.0005		82.5
10E,12Z-Octadecadienoic acid	2254177	4066172	1.80	0.85	0.0070		
α-Eleostearic acid	423818938	762389661	1.80	0.85	0.0124		88.4
5-Aminosalicylic Acid	13835434	24474492	1.77	0.82	0.0018		
cis-7-Hexadecenoic Acid	4425046	7813509	1.77	0.82	0.0322		
Lauric acid ethyl ester	324049247	537858113	1.66	0.73	0.0122		91.5
methyl 3,4,5-trihydroxycyclohex-1-ene-1-carboxylate	726765	1202260	1.65	0.73	0.0474		68.9
Progesterone	4205948	6947449	1.65	0.72	0.0376	62.7	81.9
ethyl 4-({3-[5-(trifluoromethyl)-2-pyridyl]prop-2-ynyl}oxy) benzoate	533238	876221	1.64	0.72	0.0409		66.6
5-Hydroxyindole	1149375	1812789	1.58	0.66	0.0288		
16,16-Dimethyl prostaglandin A2	802925	1235142	1.54	0.62	0.0373		71.4
O1-[4-(tert-butyl)benzoyl]-2-(tert-butylsulfonyl)ethanehydroximamide	4957848	7563783	1.53	0.61	0.0183		43.6
(R)-3-Hydroxy myristic acid	1577426	2398092	1.52	0.60	0.0122		54.8
L-Ascorbate	26634310	40178887	1.51	0.59	0.0313		
2-morpholino-1-phenyl-1-ethanol	799092	1200277	1.50	0.59	0.0241		66.2

6-Methylnicotinamide	6506153	9755016	1.50	0.58	0.0345	80.7	83.6
β-Cortolone	3869930	5797834	1.50	0.58	0.0365		87.3
Thiamine	546313	800181	1.46	0.55	0.0063		43.5
Corticosterone	80360115	114633150	1.43	0.51	0.0065	84	92.6
5-Methyluridine	4170233	5814861	1.39	0.48	0.0241		
3-(3,4-Dihydroxyphenyl)-2-Methylalanine	3559640	4864396	1.37	0.45	0.0377		
Desoxycortone	3647557	4981599	1.37	0.45	0.0484		91.1
LPC 16:3	1402985	1901338	1.36	0.44	0.0329	59.8	
Delta-Tridecalactone	2215830	2983743	1.35	0.43	0.0065		
6-(3-hydroxybutan-2-yl)-5-(hydroxymethyl)-4-methoxy-2H-pyran-2-one	1058311	1406012	1.33	0.41	0.0295		52.6
3-(3,4,5-trimethoxyphenyl)propanoic acid	670997	887345	1.32	0.40	0.0395		58
(5S)-5-hydroxy-1,7-diphenylheptan-3-one	1838826	2372104	1.29	0.37	0.0286		46.8
11(Z),14(Z)-Eicosadienoic acid	158052211	201530127	1.28	0.35	0.0527		74.4
4-(anilinomethylidene)-3-methyl-4,5-dihydroisoxazol-5-one	4298345	5472674	1.27	0.35	0.0186		47.8
HET0016	3322561	4126736	1.24	0.31	0.0078		66.1
Sedanolide	1543626	1857925	1.20	0.27	0.0015		49.8
DL-Malic acid	97285019	25315203	0.26	-1.94	0.0315	86.6	73.6
N-lactoyl-phenylalanine	6047597	2167332	0.36	-1.48	0.0460		
Acetylcarnitine	7252373	2940359	0.41	-1.30	0.0301		
L-(+)-Tartaric acid	14219773	6092814	0.43	-1.22	0.0345	68.5	70.4
3-Phosphoglyceric acid	6881588	3134113	0.46	-1.13	0.0056		44.7
D-Sphingosine	10678027	5079305	0.48	-1.07	0.0131		66.2
4-morpholinobenzoic acid	541531	258654	0.48	-1.07	0.0240		55
Fumaric acid	29379414	14535381	0.49	-1.02	0.0473	50.9	86.8
N-Acetylaspartylglutamic acid	179207	89190	0.50	-1.01	0.0344	53.7	
Kynurenic acid	23172695	11727075	0.51	-0.98	0.0536	93	90.9
PS (18:0/18:2)	1186786	606203	0.51	-0.97	0.0037	58.9	
2-Amino-1,3-octadecanediol	6829193	3521307	0.52	-0.96	0.0170	92.5	85.4
L-Anserine	14311061	7404429	0.52	-0.95	0.0045	90.9	90.7
N1-(4-chlorophenyl)-2-cyano-4,4-dimethyl-3-oxopentanamide	26610907	13840536	0.52	-0.94	0.0513		41
Pentacosanoic acid	1375043	735936	0.54	-0.90	0.0082		42.1

D-Glucose 6-phosphate	14466601	8146978	0.56	-0.83	0.0029	87.5	83.9
PI (19:0/20:4)	3431669	1938549	0.56	-0.82	0.0096	50.5	
Heneicosanoic acid	1437822	825742	0.57	-0.80	0.0119	54.5	
Urethane	105522316	62210000	0.59	-0.76	0.0008		
N-Acetyl-α-D-glucosamine 1-phosphate	1709446	1042761	0.61	-0.71	0.0298	76.5	73.8
4-Pyridoxic acid	1634478	999593	0.61	-0.71	0.0121	71.5	76.6
N-Acetyl-L-leucine	48218020	30364057	0.63	-0.67	0.0263		41.9
4-Hydroxymandelonitrile	21827256	13771729	0.63	-0.66	0.0261	60.7	
Citric Acid	602423433	382935803	0.64	-0.65	0.0083		
Dl-Proline	218396152	139454631	0.64	-0.65	0.0033		
DL-4-Hydroxyphenyllactic acid	5215720	3373504	0.65	-0.63	0.0355		44.5
PC (17:0/18:2)	1437317	947780	0.66	-0.60	0.0362	62.7	
L-Lysine	112200668	74013278	0.66	-0.60	0.0059	51.2	
Citraconic acid	94511470	62597606	0.66	-0.59	0.0190	92.1	72.6
Allantoin	14108724	9425726	0.67	-0.58	0.0260		
Norethisterone	3191964	2187159	0.69	-0.55	0.0105		
Lignoceric acid	6333219	4344650	0.69	-0.54	0.0066		60.6
Tretinoin	9076854	6228867	0.69	-0.54	0.0353	52.1	77.2
L-Tryptophan	2037219	1398142	0.69	-0.54	0.0470	58.3	47.8
L-Cysteine-glutathione gisulfide	15702325	10926658	0.70	-0.52	0.0320		
PC (15:0/15:0)	3957056	2759839	0.70	-0.52	0.0165	74.2	
Dihydrouracil	8361669	5887580	0.70	-0.51	0.0360		
Pantothenic acid	29302647	20695519	0.71	-0.50	0.0209		
Methionine	38496320	27310152	0.71	-0.50	0.0423	89.7	79.5
4-Phenylbutyric acid	2023236	1438002	0.71	-0.49	0.0235	58.3	80.3
L-Glutamic acid	18959313	13628376	0.72	-0.48	0.0228	82.7	77.6
Retinoic acid	5000913	3640242	0.73	-0.46	0.0514	61.4	
LPA 18:2	35372987	25768081	0.73	-0.46	0.0465	95.9	
Prolylleucine	88291469	64568003	0.73	-0.45	0.0118		76.4
Kojic acid	66898755	49178211	0.74	-0.44	0.0487		68.8
Creatinine	31688377	23298632	0.74	-0.44	0.0179		
Pyridoxine	2883656	2122102	0.74	-0.44	0.0347		
PC (18:5e/19:2)	1340753133	994304596	0.74	-0.43	0.0108	53.1	
PI (18:0/22:6)	10319125	7802085	0.76	-0.40	0.0321	53.2	
DL-Lysine	52305380	39547085	0.76	-0.40	0.0141	84.8	76.1
Pipecolic acid	51857911	39239890	0.76	-0.40	0.0147	84	79
2-Amino-1,3,4-octadecanetriol	619919	470261	0.76	-0.40	0.0181	61	67.8
PC (20:3/20:4)	95439864	72408001	0.76	-0.40	0.0191	67.3	

17α-Ethinylestradiol	1089750	837828	0.77	-0.38	0.0365		
LPI 20:3	3788679	2939968	0.78	-0.37	0.0371	57.9	
PC (18:5e/17:2)	7089262	5643721	0.80	-0.33	0.0181	57.6	
LPG 22:6	3113207	2534938	0.81	-0.30	0.0265	74	
LPA 20:4	16119115	13388374	0.83	-0.27	0.0356	94.3	
2-Isopropylmalate	42118059	35024192	0.83	-0.27	0.0429		

PBS is HFD+PBS group; LBC is HFD+LBC group; FC: fold change of LBC/PBS.

LBC: Live *Blautia coccoides*

mzCloud and mzValut best match score ranges from 0 to 100 (0 lowest and 100 highest).

The higher score, the higher confidence.

Table S2. The genes of I3AA-producing and leucine biosynthesis from the *B. coccoides* GA1 genome

Gene ID	Gene name	Gene description	Location	Length (bp)
gene2035	*ArAT-I*	Aromatic amino acid aminotransferase	Scaffold9	1191
gene3081	*iorA*	Indolepyruvate ferredoxin oxidoreductase subunit alpha	Scaffold16	1740
gene3082	*iorB*	Indolepyruvate ferredoxin oxidoreductase subunit beta	Scaffold16	573
gene3067	*AO*	Aldehyde oxidase	Scaffold16	2310

Table S3. Characteristics of study participants

Indices	HC	MS	P value
Samples	43	78	/
Sex（F/M)	27/16	49/29	/
Age (years)	29.56 ± 4.87	31.87 ± 9.70	0.1459
Body weight (kg)	56.48 ± 9.21	111.74 ± 26.48	< 0.0001
BMI (kg/m^2)	20.48 ± 2.12	40.86 ± 11.19	< 0.0001
Waist (cm)	72.51 ± 8.0	123.31 ± 17.65	< 0.0001
Fasting blood glucose (mmol/L)	4.57 ± 0.35	6.33 ± 2.52	< 0.0001
Fasting insulin (mIU/L)	6.92 ± 2.18	24.48 ± 13.00	< 0.0001
HOMA-IR	1.40 ± 0.44	6.85 ± 4.37	< 0.0001
HbA1c (%)	5.04 ± 0.37	6.18 ± 1.21	< 0.0001
ALT (U/L)	22.87 ± 24.49	58.26 ± 57.20	0.0002
AST (U/L)	20.61 ± 8.18	35.20 ± 26.64	0.0007
TC (mmol/L)	4.73 ± 0.90	5.04 ± 0.90	0.0807
TG (mmol/L)	1.18 ± 1.33	1.73 ± 0.83	0.0063
HDL (mmol/L)	1.52 ± 0.30	1.04 ± 0.27	< 0.0001
LDL (mmol/L)	2.84 ± 0.79	3.15 ± 0.62	0.0218

All values are expressed as the mean ± SD. Statistical comparisons were performed by nonparametric Mann-Whitney *U* test. BMI, body mass index; HOMA-IR, homeostasis model assessment of insulin resistance; HbA1c, hemoglobin A1c; ALT, alanine aminotransferase; AST, aspartate aminotransferase; TC, total cholesterol; TG, triglyceride; HDL, high-density lipoprotein LDL, low-density lipoprotein.

Table S4. Composition of the diets

Diet	Control Diet		Leucine-deprived Diet	
	gm %	kcal %	gm %	kcal %
Protein	17	17.6	16.4	17.0
Carbohydrate	68.5	70.8	69.1	71.4
Fat	5	11.6	5	11.6
kcal/gm		3.87		3.87
L-Arginine	10	40	10	40
L-Histidine	6	24	6	24
L-Isoleucine	8	32	8	32
L-Leucine	12	48	0	0
L-Lysine-HCl	14	56	14	56
L-Methionine	6	24	6	24
L-Phenylalanine	8	32	8	32
L-Threonine	8	32	8	32
L-Tryptophan	2	8	2	8
L-Valine	8	32	8	32
L-Alanine	10	40	10	40
L-Asparagine-H2O	5	20	5	20
L-Aspartate	10	40	10	40
L-Cystine	4	16	4	16
L-Glutamic Acid	30	120	30	120
L-Glutamine	5	20	5	20
Glycine	10	40	10	40
L-Proline	5	20	5	20
L-Serine	5	20	5	20
L-Tyrosine	4	16	4	16
Total L-Amino Acids	170	0	158	0
Corn Starch	550.5	2202	562.5	2250
Maltodextrin-10	125	500	125	500
Cellulose	50	0	50	0
Corn Oil	50	450	50	450
Mineral Mix S10001	35	0	35	0
Sodium Bicarbonate	7.5	0	7.5	0
Vitamin Mix V10001	10	40	10	40
Choline Bitrartrate	2	0	2	0
Red Dye, FD&C #40	0	0	0.025	0
Blue Dye, FD&C #1	0.05	0	0	0
Yellow Dye, FD&C #5	0	0	0.025	0
Total	1000.05	3872	1000.05	3872

Table S5. LC-MS/MS parameters for targeted metabolites

Instrument	4000 Q TRAP LC-MS/MS (AB Sciex)			
Chromatographic column	Thermo Hypersil Gold 2.1mm × 100mm, 3 µm			
Mobile phases	A is 0.1% formic acid aqueous solution; B is 0.1% formic acid acetonitrile solution			
Chromatographic elution gradient				
Time (min)	Flow rate (ul/min)	A %	B %	
0	200	90	10	
3	200	10	90	
8	200	10	90	
8.5	200	90	10	
15	200	90	10	
Sample injection	10ul			
Mass spectrum parameters	IS, 5500; TEM, 500 degree; GS1, 50; GS2, 50; Positive ion scanning			
Compounds	Q1/Q3	DP	CE	CXP
Indole-3-acetic acid	176.1/130.1	70	20	10
Tryptophan	205.2/188.2	45	15	10
Indole	118.1/91.1	67	32	7
3-indoleacrylic acid	188.1/170.1	63	18	9
indole-3-carboxaldehyde	146.1/118.1	68	21	9
indole-3-lactic acid	206.1/160.1	70	17	8

Table S6. qRT-PCR primers

Primers	Forward primer (5'-3')	Reverse primer (5'-3')
Ucp1	ACTGCCACACCTCCAGTCATT	CTTTGCCTCACTCAGGATTGG
Cidea	TGCTCTTCTGTATCGCCCAGT	GCCGTGTTAAGGAATCTGCTG
Prdm16	CAGCACGGTGAAGCCATTC	GCGTGCATCCGCTTGTG
Pgc1a	GATGGCACGCAGCCCTAT	CTCGACACGGAGAGTTAAAGGAA
Ahr	TGTGCAGAATCCCACATCCG	AATCAAGCGTGCATTGGACTG
Cyp1a1	GGGTTTGACACAGTCACAACT	GGGACGAAGGATGAATGCCG
Cyp1b1	ACGACGATGCGGAGTTCCTA	CGGGTTGGGAAATAGCTGC
GAPDH	TGTGTCCGTCGTGGATCTGA	CCTGCTTCACCACCTTCTTGAT
